# Alterations in bone marrow metabolism are an early and consistent feature during the development of MGUS and multiple myeloma

**DOI:** 10.1038/bcj.2015.85

**Published:** 2015-10-16

**Authors:** C Ludwig, D S Williams, D B Bartlett, S J Essex, G McNee, J W Allwood, E Jewell, A Barkhuisen, H Parry, S Anandram, P Nicolson, C Gardener, F Seymour, S Basu, W B Dunn, P A H Moss, G Pratt, D A Tennant

**Affiliations:** 1 School of Cancer Sciences, College of Medical and Dental Sciences, University of Birmingham, Birmingham, UK; 2 School of Biosciences, College of Life and Environmental Sciences, University of Birmingham, Birmingham, UK; 3 Department of Haematology, The Royal Wolverhampton Hospitals NHS Trust, Wolverhampton, UK; 4 Department of Haematology, Birmingham Heartlands Hospital, Birmingham, UK

Multiple myeloma (MM) is a malignant disorder of plasma cells arising from a clinically benign state known as monoclonal gammopathy of undetermined significance (MGUS). The biological mechanisms for disease progression are not well understood. As acquisition of malignant phenotypes by a tumour is often associated with metabolic transformation, the cancer metabolome may act as a dynamic read-out of its phenotype. The aim of this investigation was therefore to characterise the metabolic profile within the bone marrow of healthy donors and patients with MGUS or MM, and to determine if any observed differences between these disorders could be observed within peripheral plasma.

MM is a tumour of the bone marrow characterised by bone destruction, anaemia, renal impairment and infection. It remains incurable despite significant improvements in therapy. MM is an extremely heterogeneous disease both genetically and clinically with variability in presentation, response to treatment, duration of remissions and overall survival.^1^ However, it almost invariably develops from the asymptomatic premalignant stage, MGUS.^2^ MGUS is common in the elderly but only a minority of MGUS individuals progress to develop symptomatic MM, with a frequency of progression of around 1% per year. Interestingly, no specific molecular changes have yet been identified as associated with the progression of MGUS to MM: the genomic architecture of both diseases being remarkably similar.^3^ The drivers that underlie the development of MGUS and subsequent progression of patients to malignant MM are therefore largely unknown. Because of the large number of potential players within the bone marrow niche in which the disease develops, it is possible that the driver may not necessarily be the transformed plasma cell, but rather a supporting cell type, or microenvironment. Metabolic profiling of the niche in which MGUS and MM inhabits may therefore shed light on the biological processes underpinning this disease. However, there have been few studies characterising the metabolic microenvironment of MM, and to our knowledge, none on patients with MGUS. We therefore investigated the metabolome of the bone marrow niche using filtered plasma derived from bone marrow aspirates of control, MGUS and MM patients by ^1^H-NMR spectroscopy (patient cohort information; Supplementary Table 1, example annotated spectrum; Supplementary Figure 1a, for methods; Supplementary Methods). Principal component analysis (PCA) showed that the different patient groups could be separated based on the metabolite peaks detected using two components: PC1 and PC5 (Figure 1a and Supplementary Methods). Importantly, PC1 alone, which contained over 50% of the total variance in the sample, separated control samples from MGUS and MM. In contrast, MGUS and MM were only separated in PC5, which contained very little (2.7%) of the variance. This suggested that the majority of changes in the metabolites that we detected in the bone marrow of patients occur with the development of MGUS and not with the progression to MM.

We therefore investigated those metabolic changes that were associated with development of the disease. Of the metabolite peaks that were identified, the essential amino acids, isoleucine and threonine were found to be significantly decreased in bone marrow of MGUS and MM patients as were the nucleotide-breakdown products, hypoxanthine and xanthine (Figure 1b). As essential amino acids are only available from exogenous sources, these changes suggest an increase in the anabolism of the cells occupying the bone marrow in MGUS and MM, as would be expected bearing in mind the proliferation of plasma cells in this niche. In addition, excretion of both creatine and urea were significantly changed in this niche, with urea production increasing over fourfold and creatine export decreasing over 16-fold (Figure 1b). These metabolites are both products of arginine metabolism in the urea cycle, suggesting that this pathway is significantly altered in the bone marrow of MGUS and MM patients. Importantly, the significant increase observed in urea in MGUS patients is unlikely to be because of renal failure, as urine creatinine levels were only elevated in a sub set of patients (Supplementary Table 1). More work is needed to identify the driver(s) of this change in metabolism and the cell type in which it occurs. Interestingly, lactate concentrations did not significantly alter between any of the groups (Figure 1b). Lactate is considered to be a marker of transformed metabolism, and indeed its increased production in normoxia is the defining feature of the Warburg Effect. The absence of a significant increase in lactate concentrations may be owing to either efficient excretion from the bone marrow niche, or similarly to some normal tissues and solid cancers a metabolic synergy between the different cell types or microenvironments within the tumour that allows the lactate to be metabolised.^4, 5, 6^

The metabolism of hypoxanthine to xanthine occurs in the extracellular space, and metabolises molecular oxygen to form hydrogen peroxide. As concentrations of both metabolites changed in the MGUS and MM samples compared with control (Figure 1b), we used the ratio of xanthine to hypoxanthine as a correlate for the production of peroxide in the bone marrow microenvironment. The increased ratio observed in MGUS and MM samples suggested that this niche becomes more oxidative with disease (Figure 1c). This more oxidative environment is likely capable of eliciting new mutational events, and consistent with the increased oxidative stress observed in patients with MM.^7^ Our data suggest that this is also present in MGUS patients, and that dysregulated bone marrow redox may be a unifying phenotype that drives the development of paraproteinaemia.

As we were able to detect significant alterations in metabolites in the bone marrow microenvironment, we investigated whether similar changes could be observed in the peripheral plasma (patient cohort information: Supplementary Table 2). PCA analysis of the ^1^H-NMR spectra derived from this biofluid showed that although the control group clustered, the MGUS and MM patient samples demonstrated a much more heterogeneous pattern (Figure 1d). In view of this, we chose metabolites for analysis that we had previously identified as being altered in the bone marrow (Supplementary Figure 1b). However, in the peripheral plasma, none of the previously identified metabolites detected were significantly altered, whereas creatine concentrations were too low to be detected using this approach. We therefore subjected peripheral plasma samples from the same cohort to metabolomic analysis using the more sensitive ultra-high performance liquid chromatography-mass spectrometry (Supplementary Methods). PCA was again used to investigate whether the metabolites identified were able to cluster the samples into their groups. We found that control patients separated from MGUS and MM patients in PC1 for the positively charged ion data set and in PC2 for the negatively charged ion data set (Figures 2a and b), with a greater separation in the former. For the positively charged ion data set we also observed some partial separation of MM patients from MGUS patients (Figure 2a), though this was not a clear separation as observed for distinguishing control patients from MGUS and MM patients, consistent with the results from the bone marrow data set (Figure 1a). After statistical analysis, these observations were reflected in the number of metabolites that separated the groups, with over 200 putatively annotated metabolites separating control from both MGUS and MM (Supplementary Tables 3 and 4), and only 26 differentially detected between MGUS and MM (*P*<0.005; Supplementary Table 5). A number of metabolite classes were over-represented in the putative metabolites identified that varied between control, MGUS and MM samples (Figures 2c–f). As many of the classes are associated with lipid metabolism, this suggests the dysregulation of this area of metabolism may have a role in disease pathogenesis or be useful as biomarkers, and that future studies to investigate these pathways in the bone marrow, and specifically plasma cells, of patients with MGUS and MM are likely to be revealing.

We therefore show that MGUS and MM patients can be differentiated from healthy individuals using both bone marrow and peripheral plasma. In addition, we demonstrate that the metabolites that were detected vary more between control and ‘disease' than between patients with MGUS and MM. This has important implications for our understanding of this disease, as it suggests that there is an early metabolic transformation of the bone marrow microenvironment that may not be dependent on significant plasma cell occupancy, and that the progression of the disease to MM requires changes to the metabolic phenotype that are not necessarily visible in this niche. This hypothesis is also supported by recent genetic studies that suggest that there are few genetic changes associated with progression from MGUS to MM.^8^

Metabolomic approaches can be used to identify biomarkers and abnormal biological pathways in a number of diseases in a wide range of contexts.^9, 10, 11, 12, 13^ However, as the metabolome can be significantly altered by factors such as gender, age and body mass index, care must be taken through use of controlled cohorts and unbiased analysis methods. There are currently very limited data in MM patients with only a small number of publications to date,^11, 13, 14, 15, 16^ although more studies have been performed using myeloma cell lines. These studies have predominantly focused on changes following therapy or changes in individual patients at different time points in their disease, while this study is the first to compare global metabolites in MM, MGUS and healthy controls. Our study, as well as those of others, suggests that peripheral serum or plasma may well represent a means of determining disease status for patients with MGUS and MM.^14, 15, 16^ In addition, delineation of the characteristic metabolic abnormalities that underlie the development of paraproteinaemia may reveal novel opportunities for therapeutic intervention.

## Figures and Tables

**Figure 1:**
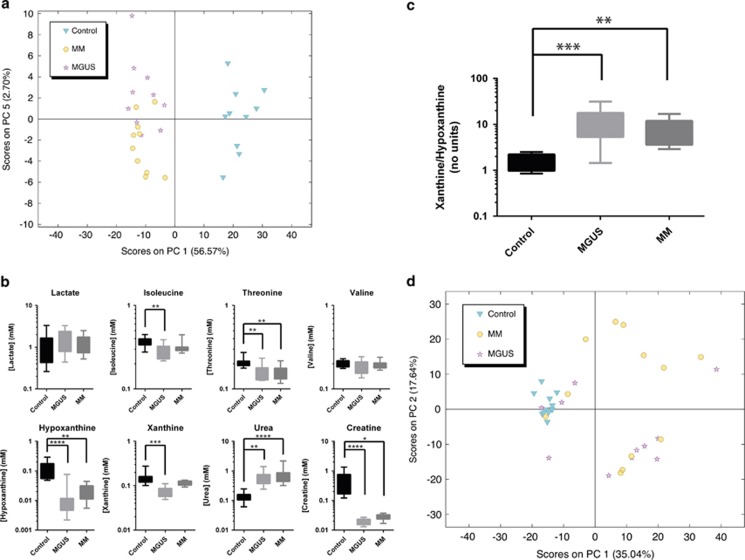
NMR spectroscopy profiling of the metabolome of the bone marrow niche of patients with MGUS and MM. (**a**) Unbiased multivariate PCA of the ^1^H spectra demonstrated significant separation of the bone marrow samples using two components: PC1 and PC5. PC1, which separated control samples from both MGUS and MM contained almost 57% of the variation. In total, 10 samples from each group were analysed. (**b**) Quantification of the labelled metabolites through back-calculation of their concentrations that contributed to the loadings in PC1 are shown. Data are represented by box and whisker plots with min–max whiskers. A non-parametric ANOVA (Kruskal–Wallis) test was used to test for significance, and where a significant change was observed, a Dunn's multiple comparisons *post hoc* test was performed. For ANOVA, *P*=0.0072 (isoleucine), 0.0043 (threonine), 0.0001 (hypoxanthine), 0.0001 (xanthine), <0.0001 (urea), <0.0001 (creatine). Where shown: *P<0.05, **P<0.01, ***P<0.001, ****P<0.0001 by Dunn's multiple comparisons test. (**c**) Ratiometric analysis of xanthine/hypoxanthine as a correlative measure of extracellular peroxide production in the bone marrow shows that it is increased with disease. ANOVA (Kruskal–Wallis) test=0.0003, Dunn's multiple comparisons test performed, * values as shown above. (**d**) PCA analysis of the ^1^H spectra acquired from peripheral plasma shows little separation between different patient groups.

**Figure 2:**
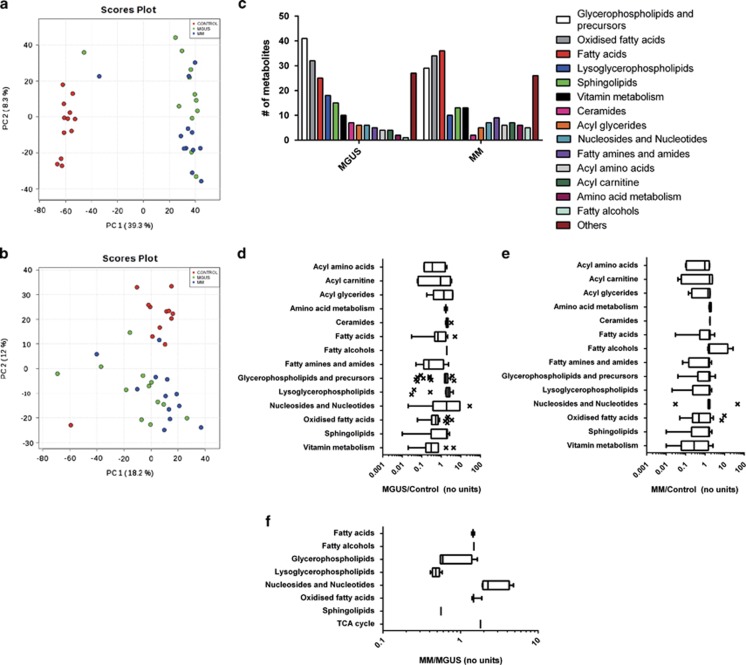
Mass spectrometry profiling of the peripheral plasma metabolome of patients with MGUS and MM. (**a** and **b**) PCA analysis of the UHPLC-MS positive (**a**) and negative (**b**) ion mode data acquired from peripheral plasma shows separation of healthy controls from MGUS and MM patients and no clear separation of MGUS patients from MM patients. (**c**) Overview of the metabolite classes that were significantly altered (cut-off *P*<0.005) in the peripheral plasma with respect to control samples. (**d**) Quantification of the relative change in each metabolite class in MGUS compared with healthy controls. Box and whisker plots show Tukey's Whiskers with outliers marked. (**e**) Quantification of the relative change in each metabolite class in MM compared with healthy controls. (**f**) Quantification of the relative change in each metabolite class in MM compared with MGUS samples. Abbreviation: TCA, tricarboxylic acid.
