# Supplementary figures and images for: Alterations in bone marrow metabolism are an early and consistent feature during the development of MGUS and multiple myeloma
Source: Blood Cancer J. 2015 Oct 16;5(10):e359–. doi: 10.1038/bcj.2015.85 (PMC4635194; doi:10.1038/bcj.2015.85)

a

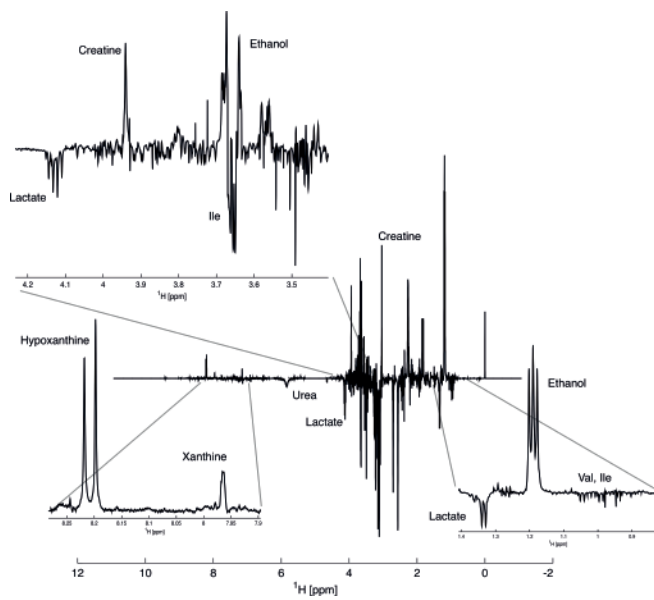

b

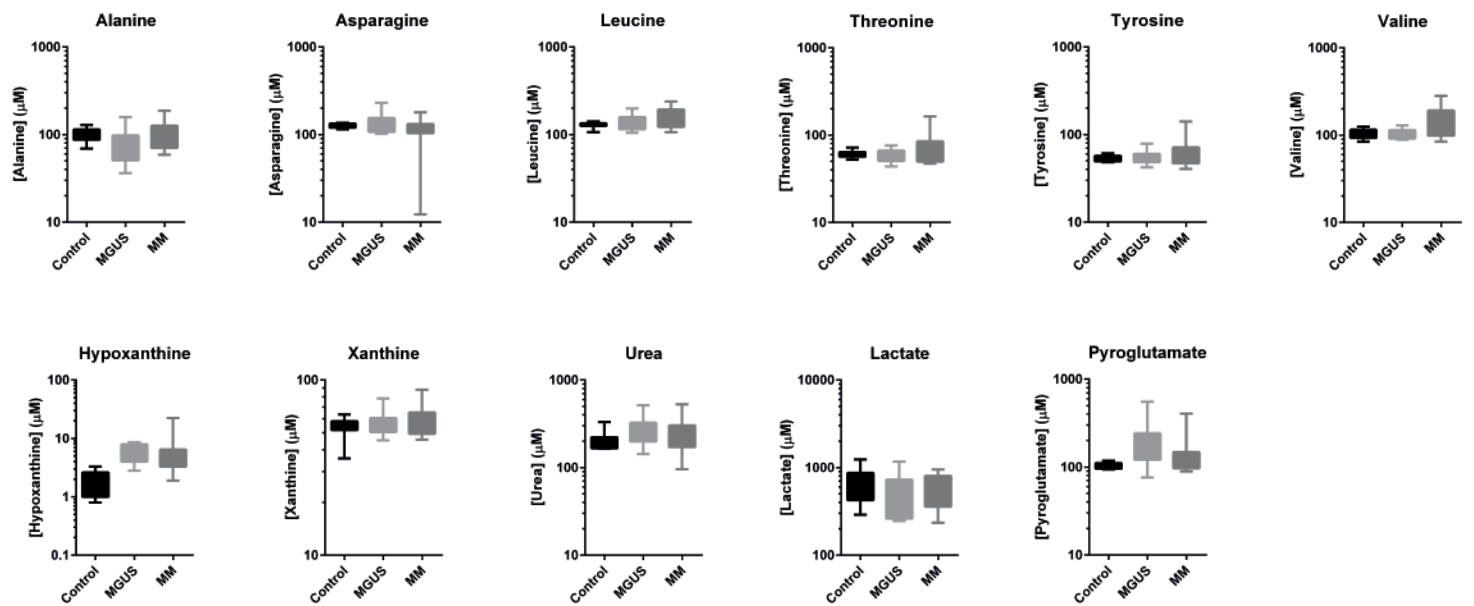

Supplementary Figure 1

Supplement: Supplementary Figure 1 [file bcj201585x1.pdf]
