# Supplementary material for: Alterations in bone marrow metabolism are an early and consistent feature during the development of MGUS and multiple myeloma
Source: Blood Cancer J. 2015 Oct 16;5(10):e359–. doi: 10.1038/bcj.2015.85 (PMC4635194; doi:10.1038/bcj.2015.85)
Supplement: Supplementary Table 1 [file bcj201585x2.docx]

**Supplementary Table 1: Patient cohort used for bone marrow plasma study**

**Cohort mean age:**

CONTROL: 67.8 years

MGUS: 65.4 years

MM: 70.1 years

**1a: MM patients**

| **Patient ID** | **Age** | **Sex** | **PP type and level** | **Plasma cells (%)** | **Creatinine** | **B2M** | **Albumin** | **Stage (ISS)** |
| --- | --- | --- | --- | --- | --- | --- | --- | --- |
| HT001 | 72 | M | IgA/k 42g/L | 38% | 80 | 3.2 | 37 | 1 |
| HT002 | 71 | M | IgG/l 30g/L | 30% | 133 | 6 | 31 | 3 |
| HT003 | 89 | F | IgG/k 32g/L | 20% | 92 | 6.11 | 34 | 3 |
| HT004 | 88 | M | IgA/k 7g/L | 26% | 236 | N.D. | 36 | N.D. |
| HT005 | 55 | M | IgG/k 25g/L | 25-30% | 119 | 5.44 | 38 | 2 |
| HT006 | 57 | M | IgG/l 83g/L | 80-90% | 110 | 15.1 | 25 | 3 |
| HT007 | 68 | F | IgG/l 19g/L | 10-15% | 91 | 3.54 | 39 | 2 |
| HT008 | 68 | M | IgA/k 15g/L | >10% | 108 | 3.4 | 38 | 1 |
| HT009 | 49 | F | IgG/k 39g/L | 75% | 90 | 3.2 | 35 | 1 |
| HT010 | 84 | M | SFLC l4140 | 30% | 276 | 33.2 | 32 | 3 |

**1b: MGUS patients**

| **Patient ID** | **Age** | **Sex** | **PP type and level** | **Plasma cells (%)** | **sFLC ratio (normal or abnormal)** | **Creatinine** | **Progression in 2 years?** |
| --- | --- | --- | --- | --- | --- | --- | --- |
| HT011 | 51 | F | IgG/L, 4g/L | 3% | Normal | 64 | No |
| HT012 | 52 | F | IgG/L, 2g/L | <1% | Normal | 63 | No |
| HT013 | 71 | M | IgA/L, N.D. | <1% | Normal | 145 | No |
| HT014 | 65 | M | IgG/L, 2g/L | <5% | Normal | 74 | No |
| HT015 | 81 | M | IgG/K, 5g/L | <1% | Normal | 98 | No |
| HT016 | 63 | M | IgG/K, 2g/L | <1% | Abnormal | 100 | No |
| HT017 | 78 | F | IgG/K, 6g/L | <5% | Abnormal | 65 | No |
| HT018 | 59 | M | IgG/L, 11g/L | 2% | Abnormal | 92 | No |
| HT019 | 59 | M | IgG/K, 14g/L | <5% | Abnormal | 228 | No |
| HT020 | 75 | M | IgG/L, 12g/L | 6% | Normal | 206 | No |
