# Supplementary material for: Alterations in bone marrow metabolism are an early and consistent feature during the development of MGUS and multiple myeloma
Source: Blood Cancer J. 2015 Oct 16;5(10):e359–. doi: 10.1038/bcj.2015.85 (PMC4635194; doi:10.1038/bcj.2015.85)
Supplement: Supplementary Table 2 [file bcj201585x3.docx]

**Supplementary Table 2: Patient cohort used for peripheral plasma study**

**Cohort mean age:**

CONTROL: 74.9 years

MGUS: 72.4 years

MM: 64.8 years

**2a: MM patients**

| **Patient ID** | **Age** | **Sex** | **PP type and level** | **Plasma cells (%)** | **Creatinine** | **B2M** | **Albumin** | **Stage (ISS)** |
| --- | --- | --- | --- | --- | --- | --- | --- | --- |
| NX001 | 65 | F | IgG/l | 25% | 64 | 2.77 | 40 | 1 |
|  |  |  | 9 g/L |  |  |  |  |  |
| NX002 | 75 | M | IgA/K | 65% | 101 | 3.05 | 31 | 2 |
|  |  |  | N.D. |  |  |  |  |  |
| NX005 | 65 | F | IgG/K | 90% | 66 | 3.96 | 33 | 2 |
|  |  |  | 36 g/L |  |  |  |  |  |
| NX006 | 63 | M | IgG/L | 20% | 73 | 2.44 | 37 | 2 |
|  |  |  | 13 g/L |  |  |  |  |  |
| NX012 | 75 | M | IgA/L | 40% | 201 | 6.22 | 32 | 3 |
|  |  |  | 9 g/L |  |  |  |  |  |
| NX013 | 67 | M | IgA/K | N.D. | 126 | 5.4 | 32 | 2 |
|  |  |  | 7 g/L |  |  |  |  |  |
| NX016 | 55 | F | IgG/L | 30% | 66 | 2.01 | 33 | 2 |
|  |  |  | 9 g/L |  |  |  |  |  |
| NX017 | 73 | F | IgG/K | 50% | 73 | 4.93 | 27 | 2 |
|  |  |  | 36 g/L |  |  |  |  |  |
| NX027 | 58 | M | IgG/K | 50% | 66 | 2.77 | 31 | 2 |
|  |  |  | 20 g/L |  |  |  |  |  |
| NX030 | 69 | F | IgA/K | 10% | 73 | 3.44 | 43 | 1 |
|  |  |  | 2.89 g/L |  |  |  |  |  |
| HT021 | 48 | M | IgG/K | 26% | 86 | 2.3 | 39 | 1 |
|  |  |  | 37 g/L |  |  |  |  |  |

**2b: MGUS patients**

| **Patient ID** | **Age** | **Sex** | **PP type and level** | **Plasma cells (%)** | **sFLC ratio (normal or abnormal)** | **Creatinine** | **Progression at 2 years?** |
| --- | --- | --- | --- | --- | --- | --- | --- |
| NX003 | 76 | M | IgG/L, 2 g/L | <1% | Abnormal | 88 | No |
| NX008 | 75 | M | IgG/K, 11 g/L | 9% | Normal | 97 | No (RIP at 18 months) |
| NX009 | 72 | M | IgG/L, 8 g/L | 6% | Abnormal | 75 | No |
| NX010 | 70 | F | IgG/K, 2 g/L | <1% | Normal | 64 | No |
| NX011 | 78 | M | IgG/L, <2 g/L | <1% | Normal | 265 | No |
| NX018 | 68 | M | IgG/K, 5 g/L | <1% | Abnormal | 89 | No |
| NX019 | 83 | F | IgG/L, <2 g/L | <1% | Abnormal | 86 | RIP at 2 months |
| NX020 | 61 | M | IgG/K, 3 g/L | 1% | Abnormal | 111 | No |
| NX021 | 83 | M | IgA/L, N.D. | 9% | Abnormal | 80 | No (RIP at 21 months) |
| NX031 | 70 | F | IgG/K, 14 g/L | 6% | Abnormal | 113 | No (at 23 months) |
| NX033 | 69 | M | IgG/K, 10 g/L | 4% | Abnormal | 83 | No (at 21 months) |
| NX035 | 64 | M | IgG/K, 4 g/L | 1-2% | Abnormal | 87 | No (at 21 months) |
