# Supplementary material for: Alterations in bone marrow metabolism are an early and consistent feature during the development of MGUS and multiple myeloma
Source: Blood Cancer J. 2015 Oct 16;5(10):e359–. doi: 10.1038/bcj.2015.85 (PMC4635194; doi:10.1038/bcj.2015.85)
Supplement: Supplementary Table 3 [file bcj201585x4.docx]

**Supplementary Table 3**

Putative metabolites identified in the peripheral plasma that vary significantly (p<0.005) between control and MGUS subjects

| **m/z** | **Retention time** | **P-value** | **Fold change (control/MGUS)** | **Metabolite** | **Metabolite class** |
| --- | --- | --- | --- | --- | --- |
| 372.2293 | 193 | 0.00017 | 0.13 | N-Hexadecanoylglycine | Acyl amino acids |
| 447.7623 | 212 | 0.00017 | 0.14 | N-oleoyl taurine | Acyl amino acids |
| 172.0975 | 196 | 0.00081 | 0.55 | Hexanoylglycine;Isovalerylalanine;Isovalerylsarcosine | Acyl amino acids |
| 240.0993 | 327 | 0.00039 | 1.92 | Capryloylglycine | Acyl amino acids |
| 340.1883 | 206 | 0.00004 | 0.06 | 2,6 dimethylheptanoyl carnitine | Acyl carnitine |
| 374.2556 | 252 | 0.00053 | 0.08 | 4,8 dimethylnonanoyl carnitine | Acyl carnitine |
| 400.3425 | 298 | 0.00323 | 1.74 | Palmitoylcarnitine | Acyl carnitine |
| 460.2632 | 332 | 0.00146 | 3.31 | Tetradecenoylcarnitine | Acyl carnitine |
| 481.3487 | 347 | 0.00011 | 0.18 | MG(22:1) | Acyl glycerides |
| 661.3966 | 309 | 0.00246 | 0.47 | DG(34:5) | Acyl glycerides |
| 634.5376 | 337 | 0.00325 | 0.53 | DG(36:4) | Acyl glycerides |
| 379.2818 | 341 | 0.00066 | 2.14 | MG(18:1) | Acyl glycerides |
| 779.6516 | 267 | 0.00078 | 3.71 | DG(46:4);DG(44:1) | Acyl glycerides |
| 783.6709 | 267 | 0.00341 | 3.89 | DG(46:3) | Acyl glycerides |
| 228.0715 | 205 | 0.00003 | 1.99 | Nalpha,Nalpha-Dimethyl-L-histidine | Amino acid metabolism |
| 205.0183 | 184 | 0.00250 | 1.44 | Tryptophan | Amino acid metabolism |
| 516.3002 | 196 | 0.00034 | 0.04 | Taurocholate;Taurohyocholate;Tauroursocholic acid | Bile acids |
| 575.3170 | 323 | 0.00028 | 0.15 | Lithocholate 3-O-glucuronide | Bile acids |
| 494.2859 | 288 | 0.00489 | 0.48 | Chenodeoxyglycocholic acid;Glycochenodeoxycholic Acid;Glycodeoxycholate;Glycoursodeoxycholic acid | Bile acids |
| 491.2102 | 194 | 0.00003 | 0.04 | Octanoyl-a-D-Glucopyranosyl-B-D-Fructofuranoside | Carbohydrates |
| 331.9651 | 186 | 0.00012 | 15.67 | Ribose 1,5-bisphosphate;Ribulose 1,5-bisphosphate | Carbohydrates |
| 766.5572 | 437 | 0.00467 | 1.63 | Galactosylceramide (d18:1/18:0);Glucosylceramide (d18:1/18:0) | Ceramides |
| 806.6373 | 474 | 0.00372 | 1.77 | CerP(d18:1/26:1) | Ceramides |
| 642.5797 | 469 | 0.00098 | 1.85 | Cer(d18:1/22:1) | Ceramides |
| 668.5944 | 473 | 0.00089 | 1.97 | Cer(d18:0/22:0) | Ceramides |
| 670.6109 | 498 | 0.00221 | 2.04 | Ceramide (d18:1/24:1) | Ceramides |
| 644.5959 | 493 | 0.00458 | 2.23 | Ceramide (d18:1/22:0) | Ceramides |
| 738.5314 | 418 | 0.00082 | 3.25 | Galactosylceramide (d18:1/16:0);Glucosylceramide (d18:1/16:0) | Ceramides |
| 104.1074 | 330 | 0.00005 | 1.61 | Choline | Choline metabolism |
| 184.0730 | 330 | 0.00004 | 2.29 | Choline phosphate | Choline metabolism |
| 435.2234 | 203 | 0.00005 | 0.03 | eicosadienoic acid;Icosatrienoic acid | Fatty acids |
| 371.2199 | 194 | 0.00075 | 0.04 | Eicosapentaenoic acid;Icosapentaenoic acid | Fatty acids |
| 433.2836 | 195 | 0.00049 | 0.04 | Pentacosadiynoic acid | Fatty acids |
| 381.1946 | 208 | 0.00014 | 0.36 | Icosatrienoic acid;eicosatrienoic acid | Fatty acids |
| 209.0792 | 220 | 0.00089 | 0.37 | Azelaic acid | Fatty acids |
| 315.1547 | 204 | 0.00007 | 0.44 | Tetradecadienoic acid | Fatty acids |
| 215.1294 | 243 | 0.00053 | 0.48 | Decenoic acid | Fatty acids |
| 275.1254 | 258 | 0.00427 | 0.57 | methyl-decanoic acid | Fatty acids |
| 173.0814 | 209 | 0.00100 | 0.61 | Suberic acid | Fatty acids |
| 362.1681 | 263 | 0.00081 | 0.62 | hexadecatetraenoic acid | Fatty acids |
| 247.1191 | 203 | 0.00100 | 0.64 | Sebacic acid | Fatty acids |
| 347.2194 | 302 | 0.00194 | 0.65 | octadecatrienoic acid | Fatty acids |
| 199.0973 | 225 | 0.00389 | 0.67 | Decenedioic acid | Fatty acids |
| 249.1851 | 263 | 0.00313 | 0.69 | hexadecatrienoic acid | Fatty acids |
| 221.0818 | 242 | 0.00182 | 0.71 | Dodecatetraenedioic acid | Fatty acids |
| 295.1644 | 261 | 0.00150 | 0.71 | hexadecadienoic acid | Fatty acids |
| 261.1347 | 212 | 0.00467 | 0.71 | Undecanedioic acid | Fatty acids |
| 227.1257 | 221 | 0.00467 | 1.40 | Nonanoic acid | Fatty acids |
| 387.1817 | 340 | 0.00464 | 1.61 | methyl-hexadecanoic acid | Fatty acids |
| 247.0239 | 331 | 0.00408 | 1.77 | methyl-hexanoic acid;Heptanoic acid | Fatty acids |
| 400.2203 | 306 | 0.00458 | 1.96 | Hexadecasphinganine | Fatty acids |
| 325.2104 | 338 | 0.00017 | 2.01 | Octadecadienoic acid | Fatty acids |
| 218.2116 | 216 | 0.00081 | 2.06 | Dodecanoate | Fatty acids |
| 351.2300 | 337 | 0.00028 | 2.09 | Docosahexaenoic acid | Fatty acids |
| 255.1328 | 181 | 0.00012 | 4.98 | tridecadienoic acid | Fatty acids |
| 215.1248 | 199 | 0.00053 | 1.91 | Octanediol | Fatty alcohols |
| 282.2791 | 338 | 0.00003 | 0.05 | Elaidoylamide;Oleamide | Fatty amines and amides |
| 256.2634 | 334 | 0.00003 | 0.20 | Palmitic amide | Fatty amines and amides |
| 348.2876 | 313 | 0.00003 | 0.23 | N-Oleoylethanolamine | Fatty amines and amides |
| 316.2210 | 287 | 0.00003 | 0.27 | amino-hexadecanoic acid | Fatty amines and amides |
| 658.6110 | 508 | 0.00100 | 2.34 | Tricosanamide | Fatty amines and amides |
| 216.0628 | 198 | 0.00029 | 4.49 | Glycerylphosphorylethanolamine | Glycerophospholipid precursors |
| 855.4819 | 210 | 0.00468 | 0.05 | PG(36:3) | Glycerophospholipids |
| 833.4722 | 211 | 0.00005 | 0.06 | PG(38:6) | Glycerophospholipids |
| 899.5017 | 211 | 0.00128 | 0.06 | PI(36:2) | Glycerophospholipids |
| 943.5329 | 212 | 0.00114 | 0.09 | PIP(36:1) | Glycerophospholipids |
| 877.4975 | 212 | 0.00008 | 0.12 | PG(34:0) | Glycerophospholipids |
| 752.4270 | 208 | 0.00448 | 0.25 | PE(36:8);PE(34:5) | Glycerophospholipids |
| 876.5626 | 411 | 0.00016 | 0.25 | PI(36:4);PE(40:1) | Glycerophospholipids |
| 744.4883 | 415 | 0.00072 | 0.30 | PE(16:1/dm18:1);PE(18:2/dm16:0) | Glycerophospholipids |
| 870.5511 | 459 | 0.00053 | 1.46 | PC(20:2/dm18:1);PC(20:3/dm18:0) | Glycerophospholipids |
| 780.5510 | 432 | 0.00122 | 1.46 | PC(34:2) | Glycerophospholipids |
| 478.3285 | 432 | 0.00066 | 1.47 | PC(O-16:2/0:0) | Glycerophospholipids |
| 868.5357 | 439 | 0.00268 | 1.48 | PC(20:3/dm18:1);PC(20:4/dm18:0);PC(22:4/dm16:0) | Glycerophospholipids |
| 890.5216 | 422 | 0.00100 | 1.52 | PC(22:6/dm18:1);PE(38:1) | Glycerophospholipids |
| 824.5506 | 457 | 0.00150 | 1.52 | PE(22:4/dm18:0) | Glycerophospholipids |
| 825.5604 | 457 | 0.00077 | 1.53 | PG(40:5) | Glycerophospholipids |
| 814.5555 | 438 | 0.00467 | 1.56 | PE(36:1) | Glycerophospholipids |
| 792.5751 | 438 | 0.00221 | 1.56 | PS(36:0);PG(36:2) | Glycerophospholipids |
| 786.5991 | 458 | 0.00268 | 1.63 | PC(36:2) | Glycerophospholipids |
| 858.5947 | 452 | 0.00182 | 1.67 | PC(40:5);PC(38:2) | Glycerophospholipids |
| 809.5865 | 459 | 0.00043 | 1.70 | PC(38:5);PC(36:2) | Glycerophospholipids |
| 748.5256 | 422 | 0.00467 | 1.71 | PE(20:5/dm18:1);PE(22:6/dm16:0);PE(O-16:1/22:6);PE(18:2/dm18:1);PE(18:3/dm18:0);PE(20:3/dm16:0) | Glycerophospholipids |
| 859.5252 | 416 | 0.00256 | 1.72 | PG(38:3) | Glycerophospholipids |
| 846.5241 | 419 | 0.00034 | 1.73 | PC(36:6);PC(34:3) | Glycerophospholipids |
| 860.6114 | 472 | 0.00268 | 1.74 | PC(40:4);PC(38:1) | Glycerophospholipids |
| 771.5192 | 421 | 0.00268 | 1.76 | PG(36:4) | Glycerophospholipids |
| 764.5571 | 445 | 0.00221 | 1.86 | PC(16:1/dm18:1);PC(18:2/dm16:0) | Glycerophospholipids |
| 828.5491 | 422 | 0.00066 | 1.89 | PC(38:6);PC(36:3) | Glycerophospholipids |
| 856.5809 | 445 | 0.00221 | 1.89 | PC(42:9);PC(40:6);PC(38:3) | Glycerophospholipids |
| 835.6019 | 460 | 0.00467 | 2.01 | PC(40:6);PC(38:3);PC(36:0) | Glycerophospholipids |
| 865.5747 | 464 | 0.00014 | 2.02 | PS(38:0);PE(40:4);PE(38:1) | Glycerophospholipids |
| 871.5263 | 415 | 0.00268 | 2.08 | PC(36:5) | Glycerophospholipids |
| 836.5508 | 267 | 0.00195 | 2.18 | PC(20:4/dm18:1);PC(20:5/dm18:0);PC(22:5/dm16:0) | Glycerophospholipids |
| 740.5226 | 417 | 0.00076 | 2.18 | PE(36:4);PE(34:1) | Glycerophospholipids |
| 803.5378 | 414 | 0.00323 | 2.38 | PC(38:8);PC(36:5);PC(34:2) | Glycerophospholipids |
| 878.6586 | 267 | 0.00178 | 2.53 | PE(44:2) | Glycerophospholipids |
| 753.5215 | 413 | 0.00077 | 2.63 | PC(32:2) | Glycerophospholipids |
| 816.6510 | 267 | 0.00239 | 2.69 | PC(38:1) | Glycerophospholipids |
| 859.4888 | 268 | 0.00119 | 2.85 | PG(40:7) | Glycerophospholipids |
| 884.4720 | 268 | 0.00146 | 2.94 | PE(38:4) | Glycerophospholipids |
| 752.5186 | 413 | 0.00360 | 3.67 | PC(32:2) | Glycerophospholipids |
| 484.2426 | 199 | 0.00052 | 0.03 | LysoPC(12:0) | Lysoglycerophospholipids |
| 555.2680 | 198 | 0.00075 | 0.04 | LysoPG(18:0) | Lysoglycerophospholipids |
| 538.2892 | 215 | 0.00029 | 0.27 | LysoPC(18:4);LysoPC(16:1) | Lysoglycerophospholipids |
| 506.3606 | 458 | 0.00268 | 1.64 | LysoPC(dm18:1) | Lysoglycerophospholipids |
| 546.2574 | 321 | 0.00153 | 1.80 | LysoPE(20:4) | Lysoglycerophospholipids |
| 614.3406 | 348 | 0.00022 | 1.92 | LysoPC(18:0) | Lysoglycerophospholipids |
| 522.3551 | 336 | 0.00100 | 1.94 | LysoPC(18:1) | Lysoglycerophospholipids |
| 586.3086 | 332 | 0.00003 | 2.01 | LysoPC(18:3) | Lysoglycerophospholipids |
| 504.3071 | 328 | 0.00017 | 2.21 | LysoPE(20:3) | Lysoglycerophospholipids |
| 534.2965 | 332 | 0.00008 | 2.22 | LysoPC(16:0) | Lysoglycerophospholipids |
| 476.2765 | 328 | 0.00132 | 2.24 | LysoPE(16:0) | Lysoglycerophospholipids |
| 548.2763 | 320 | 0.00313 | 2.42 | LysoPE(22:6) | Lysoglycerophospholipids |
| 634.3098 | 324 | 0.00009 | 2.50 | LysoPC(20:4) | Lysoglycerophospholipids |
| 658.3102 | 323 | 0.00014 | 2.67 | LysoPC(22:6) | Lysoglycerophospholipids |
| 570.3539 | 329 | 0.00361 | 2.93 | LysoPC(22:5) | Lysoglycerophospholipids |
| 564.3074 | 315 | 0.00043 | 3.03 | LysoPC(20:5) | Lysoglycerophospholipids |
| 542.3223 | 325 | 0.00007 | 3.18 | LysoPC(18:2) | Lysoglycerophospholipids |
| 490.2920 | 316 | 0.00017 | 4.06 | LysoPC(14:0) | Lysoglycerophospholipids |
| 360.2766 | 280 | 0.00057 | 0.33 | 12,13-dihydroxy-11-methoxy-9-octadecenoic acid;6-hydroxysphingosine | Mixed class |
| 803.5985 | 267 | 0.00451 | 3.21 | 3-Demethylubiquinone-9;SM(d18:1/20:0) | Mixed class |
| 808.0752 | 448 | 0.00221 | 1.57 | (6S)-6-beta-Hydroxy-1,4,5,6-tetrahydronicotinamide-adenine dinucleotide 2'-phosphate | NADH metabolism |
| 791.0475 | 436 | 0.00389 | 2.13 | Deamino-NAD+ | NADH metabolism |
| 369.0067 | 70 | 0.00008 | 0.02 | Pseudouridine;Uridine | Nucleosides and nucleotides |
| 817.0502 | 422 | 0.00268 | 1.79 | 3',5'-Cyclic diGMP | Nucleosides and nucleotides |
| 214.9992 | 331 | 0.00003 | 1.90 | Dimethyluracil | Nucleosides and nucleotides |
| 563.0676 | 226 | 0.00284 | 2.30 | dTDP-galactose;dTDP-glucose | Nucleosides and nucleotides |
| 749.5391 | 418 | 0.00389 | 1.82 | CE(20:3) | Other metabolite class |
| 716.2674 | 317 | 0.00071 | 2.97 | 5-Methyltetrahydropteroyltri-L-glutamate | Other metabolite class |
| 628.2256 | 354 | 0.00499 | 0.50 | Dehydroisocoproporphyrinogen | Other metabolite class |
| 449.2181 | 209 | 0.00039 | 0.06 | Estradiol-3-glucuronide | Other metabolite class |
| 466.3232 | 352 | 0.00009 | 0.32 | 3alpha,6alpha,7alpha,12alpha-Tetrahydroxy-5beta-cholest-24-en-26-oic acid | Other metabolite class |
| 342.2484 | 354 | 0.00100 | 2.05 | 17beta-Hydroxy-2alpha,17-dimethyl-5alpha-androstan-3-one;17beta-Hydroxy-5alpha-androstane acetate;3a-Hydroxy-5b-pregnane-20-one;3-alpha-Hydroxy-5-alpha-pregnan-20-one;3alpha-Hydroxy-5beta-pregnane-20-one;3beta-Hydroxy-5alpha-pregnane-20-one;3beta-Hydroxy-5beta-pregnane-20-one | Other metabolite class |
| 225.1083 | 192 | 0.00081 | 1.95 | Dihydrolipoamide | Other metabolite class |
| 298.2472 | 309 | 0.00003 | 0.06 | Epoxyoctadecanoic acid;Hydroxyoctadecenoic acid | Oxidised fatty acids |
| 335.2552 | 325 | 0.00007 | 0.10 | oxo-nonadecanoic acid | Oxidised fatty acids |
| 337.2357 | 281 | 0.00157 | 0.12 | (13E)-(15S)-15-Hydroxy-9-oxoprosta-10,13-dienoate;"(13E)-(15S)-15-Hydroxy-9-oxoprosta-11,13-dienoate";"(5Z,8Z,10E,14Z)-(12S)-12-hydroperoxyicosa-5,8,10,14-tetraenoate";"(5Z,8Z,11Z,13E)-(15S)-15-hydroperoxyicosa-5,8,11,13-tetraenoate";"(5Z,9E,11Z,14Z)-(8R)-8-hydroperoxyicosa-5,9,11,14-tetraenoate";"(5Z,9E,14Z)-(8xi,11R,12S)-11,12-Epoxy-8-hydroxyicosa-5,9,14-trienoic acid";"(6E,8Z,11Z,14Z)-(5s)-5-hydroperoxycosa-6,8,11,14-tetraenoate";"(6z,8e,10e,14z)-(5s,12r)-5,12-dihydroxyicosa-6,8,10,14-tetraenoate";"10,11-dihydro-12-oxo-LTB4";11(R)-HPETE;"11,12-DiHETE";"11,12-dihydroxy-5(E),7(E),9(E),14(Z)-eicosatetraenoate";11-deoxy-PGE2;"11H-14,15-EETA";11-HpETE;12(R)-HPETE;12(S)-HPETE;12(S)-Leukotriene B4;"12,20-DiHETE";12R-Hydroperoxyeicosatetraenoate;"14,15-DiHETE";"14,15-dihydroxy-5,8,10,12-eicosatetraenoic acid";"14-hydroperoxy-5Z,8Z,11Z,15E-eicosatetraenoic acid";15(S)-HPETE;15-epi-PGA1;"15H-11,12-EETA";"17,18-DiHETE";20-hydroxy-5S-HETE;"5,12-dihydroxy-6,8,10,14-eicosatetraenoic acid";"5,15-DiHETE";5-HPETE;"5S,15S-DiHETE";"5S,6R-DiHETE";"5S,6S-DiHETE";"6,7-dihydro-5-oxo-12-epi-leukotriene B";6E-12-epi-leukotriene B4;"6-hydroperoxy-4E,8Z,11Z,14Z-eicosatetraenoic acid";6-trans-12-epi-Leukotriene B4;6-trans-Leukotriene B4;8(R)-HPETE;8(S)-HPETE;"8,15-DiHETE";"8,15-dihydroxy-5,9,11,13-eicosatetraenoic acid";"8,9-DiHETE";8-iso-PGA1;"8S,15S-DiHETE";"8S-hydroxy-11R,12S-epoxy-5Z,9E,14Z-eicosatrienoic acid";9(S)-HPETE;9-deoxy-delta12-PGD2;9-HpETE;Diterpenoid SP-II;Hepoxilin A3;Hepoxilin B3;Leukotriene B4;"PGF2alpha-1,11-lactone";"PGF2alpha-1,15-lactone";"PGF2alpha-1,9-lactone";Portulal;Prostaglandin A1;Prostaglandin B1;Prostaglandin C1;;"(9Z)-(7S,8S)-Dihydroxyoctadecenoic acid";"12,13-DHOME";"12,13-dihydroxy-9-octadecenoic acid";"12,13-hydroxyoctadec-9(Z)-enoate";"18-hydroxy-9R,10S-epoxy-stearic acid";"18-hydroxy-9S,10R-epoxy-stearic acid";"7S,8S-dihydroxy-9Z-octadecenoic acid";"9,10-DHOME";"9,10-dihydroxy-12-octadecenoic acid";"9,10-epoxy-18-hydroxystearate";"9,10-hydroxyoctadec-12(Z)-enoate";"9,13-dihydroxy-10-octadecenoic acid";"9,13-dihydroxy-11-octadecenoic acid";Dibutyl sebacate (NF);Octadecanedioic acid;;10E-heptadecenoic acid;10-methyl-9-hexadecenoic acid;10Z-heptadecenoic acid;14-methyl-8E-hexadecenoic acid;14R-methyl-8Z-hexadecenoic acid;14S-methyl-8Z-hexadecenoic acid;16-heptadecenoic acid;2-heptadecylenic acid;2Z-heptadecenoic acid;3-heptadecenoic acid;7-heptadecenoic acid;7-methyl-6E-hexadecenoic acid;7Z-heptadecenoic acid;8E-heptadecenoic acid;8Z-heptadecenoic acid;9E-heptadecenoic acid;9-heptadecylenic acid;omega-Cyclohexylundecanoic acid;; | Oxidised fatty acids |
| 322.2454 | 312 | 0.00003 | 0.12 | (15S)-15-Hydroxy-5,8,11-cis-13-trans-eicosatetraenoate;10-HETE;11(R)-HETE;"11,12:EpETrE";"11,12-EET";"11,12-Epoxyeicosatrienoic acid";"11beta,17beta-Dihydroxy-17-methyl-5alpha-androstan-3-one";11-HETE;"11-hydroxy-5E,8Z,12Z,14Z-eicosatetraenoic acid";11-hydroxyeicosatetraenoate;12 hydroxy arachidonic acid;12-HETE;"12-hydroxy-5Z,8Z,10Z,14Z-Eicosatetraenoic acid";12-hydroxyeicosatetraenoate;"12S-hydroxy-5E,8Z,10Z,14Z-eicosatetraenoic acid";13-HETE;"14,15-EET";"14-hydroxy-5E,8Z,11Z,15Z-eicosatetraenoic acid";"14R,15S-EpETrE";"15(R)-Hydroxy-(5Z,8Z,11Z,13E)-eicosatetraenoate";15-HETE;"15-hydroxy-5E,8Z,11Z,13Z-eicosatetraenoic acid";"15R-hydroxy-5E,8Z,11Z,13Z-eicosatetraenoic acid";16(R)-HETE;17beta-Hydroxy-2alpha-(hydroxymethyl)-5alpha-androstan-3-one;"17beta-Methoxyandrost-5-ene-3beta,16beta-diol";17-HETE;"17-Methylandrost-5-ene-3beta,11beta,17beta-triol";18 hydroxy arachidonic acid;18-HETE;19(S)-HETE;20-HETE;"20-hydroxy-5Z,8Z,11Z,14Z-eicosatetraenoic acid";3beta-Hydroxy-16beta-(hydroxymethyl)-5alpha-androstan-17-one;3R-HETE;"5,6-EET";5-HETE;"6-hydroxy-4E,8Z,11Z,14Z-eicosatetraenoic acid";7-HETE;"8,9-EET";8-HETE;"8-hydroxy-5E,9Z,11Z,14Z-eicosatetraenoic acid";8-hydroxyeicosatetraenoate;"8R-hydroxy-5E,9Z,11Z,14Z-eicosatetraenoic acid";9-HETE;"9R-hydroxy-5E,7Z,11Z,14Z-eicosatetraenoic acid";Hydroxyeicosatetraenoic acid;w-hydroxyl arachidonic acid;;"(6R,7S)-6,7-Epoxyoctadecanoic acid";(9Z)-(12S)-Hydroxyoctadecenoic acid;10-hydroxy-8-octadecenoic acid;10-keto stearic acid;10R-hydroxy-8E-octadecenoic acid;11-hydroxy-9-octadecenoic acid;11-oxo-octadecanoic acid;12-hydroxy-10E-octadecenoic acid;12-oxo-octadecanoic acid;12R-hydroxy-9E-octadecenoic acid;12S-hydroxy-9E-octadecenoic acid;12S-hydroxy-9Z-octadecenoic acid;13-oxo-octadecanoic acid;14-oxo-octadecanoic acid;15-oxo-octadecanoic acid;16-methyl-10-oxo-heptadecanoic acid;16-oxo-octadecanoic acid;17-hydroxy-9Z-octadecenoic acid;17-oxo-octadecanoic acid;18-hydroxy-9Z-octadecenoic acid;18-hydroxyoleate;2-methyl-4-oxo-heptadecanoic acid;2-Oxooctadecanoic acid;3-Oxo-Octadecanoic acid;4-keto stearic acid;5-hydroxy-2-octadecenoic acid;5-keto stearic acid;6-keto stearic acid;7-keto-stearic acid;8-hydroxy-9-octadecenoic acid;8-oxo-octadecanoic acid;8R-hydroxy-9Z-octadecenoic acid;9-hydroxy-10E-octadecenoic acid;9-hydroxy-10Z-octadecenoic acid;9-hydroxy-12-octadecenoic acid;9-hydroxy-12Z-octadecenoic acid;9-keto stearic acid;"9R,10S-epoxy-stearic acid";9R-hydroxy-10E-octadecenoic acid;9R-hydroxy-12E-octadecenoic acid;9R-hydroxy-12Z-octadecenoic acid;"9S,10R-epoxy-stearic acid";Methyl palmoxirate (USAN);Ricinelaidic acid;Ricinoleic acid;Rosaprostol (INN);;14-Methyl-8E-hexadecenal;14R-Methyl-8E-hexadecenal;14R-Methyl-8Z-hexadecenal;14S-Methyl-8E-hexadecenal;14S-Methyl-8Z-hexadecenal;14Z-Methyl-8-hexadecenal;"2,6,8,12-Tetramethyl-2,4-tridecadien-1-ol";9Z-Heptadecenal;; | Oxidised fatty acids |
| 309.0930 | 271 | 0.00050 | 0.19 | Hydroxydecanedioic acid | Oxidised fatty acids |
| 231.1239 | 220 | 0.00016 | 0.25 | hydroxy-decenoic acid;Oxodecanoate | Oxidised fatty acids |
| 319.2258 | 307 | 0.00007 | 0.27 | (+/-)-11-HEPE;(+/-)-15-HEPE;(+/-)-18-HEPE;(+/-)-8-HEPE;(+/-)-9-HEPE;"(7E,9E,11Z,14Z)-(5S,6S)-5,6-epoxyicosa-7,9,11,14-tetraenoate";11(12)-EpETE;"11-oxo-5E,8Z,12Z,14Z-Eicosatetraenoic acid";11R-HEPE;11S-HEPE;12-HEPE;"12-oxo-5Z,8Z,10E,14Z-eicosatetraenoic acid";12-OxoETE;14(15)-EpETE;15d-PGA1;15-HEPE;"15-oxo-5,8,11-cis-13-trans-icosatetraenoate";15-OxoETE;17(18)-EpETE;;"17R,18S-Epoxy-5Z,8Z,11Z,14Z-icosatetraenoic acid";"4,5-Leukotriene A4";5-HEPE;"5-oxo-(6E,8Z,11Z,14Z)-eicosatetraenoic acid";5-OxoETE;8(9)-EpETE;"8-oxo-5E,9Z,11Z,14Z-eicosatetraenoic acid";8R-HEPE;8S-HEPE;"9-hydroxy-2Z,5E,7Z,11Z,14Z-Eicosapentaenoic acid";"9-oxo-5E,7Z,11Z,14Z-eicosatetraenoic acid";9S-HEPE;"(9Z)-(12S,13R)-12,13-Epoxyoctadecenoic acid";"(9Z,12Z)-(8S)-Hydroxyoctadeca-9,12-dienoic acid";12(13)-EpOME;"12,13-epoxy-9-octadecenoic acid";12-hydroxy-10-octadecynoic acid;12-hydroxy-9-octadecynoic acid;12-oxo-10E-octadecenoic acid;12-oxo-10Z-octadecenoic acid;12-oxo-9E-octadecenoic acid;12-oxo-9Z-octadecenoic acid;"12R-hydroxy-9Z,15Z-octadecadienoic acid";12S-hydroxy-9-octadecynoic acid;13(R)-HODE;13(S)-HODE;"13-hydroxy-(9Z,11E)-octadecadienoate";"13R-hydroxy-9E,11Z-octadecadienoic acid";"13S-hydroxy-9E,11Z-octadecadienoic acid";18-oxooleate;"6-hydroxy-9Z,12Z-octadecadienoic acid";"8S-hydroxy-9Z,12Z-octadecadienoic acid";9(10)-EpOME;9(R)-HODE;9(S)-HODE;"9,10-epoxy-12-octadecenoic acid";"9,10-epoxyoctadecenoic acid";9-HODE;"9-hydroxy-10E,12Z-octadecadienoic acid";9-hydroxyoctadecadienoate;"9S-hydroxy-10E,12E-octadecadienoic acid"; | Oxidised fatty acids |
| 343.1736 | 225 | 0.00039 | 0.30 | 3-Hydroxytetradecanedioic acid | Oxidised fatty acids |
| 411.3084 | 344 | 0.00011 | 0.38 | hydroxy-heneicosanoic acid | Oxidised fatty acids |
| 379.2452 | 304 | 0.00003 | 0.44 | 13,14-dihydro PGE1;"13,14-dihydro PGF2a";"13,14-dihydro-15-keto-PGF1alpha";"13,14-dihydro-PGE1";"13,14-dihydro-PGF2alpha";PGF1beta;Prostaglandin F1alpha | Oxidised fatty acids |
| 330.2378 | 258 | 0.00017 | 0.44 | trihydroxy-octadecenoic acid | Oxidised fatty acids |
| 370.2606 | 291 | 0.00043 | 0.46 | (+/-) 5-iPF2alpha-VI;"(13E)-(15S)-11-alpha,15-dihydroxy-9-oxoprost-13-enoate";"(5Z,13E)-(15S)-9,11,15-trihydroxyprosta-5,13-dienoate";"(5Z,9E,14Z)-(8xi,11xi,12S)-8,11,12-Trihydroxyicosa-5,9,14-trienoate";"11,12,15-THETA";"11,12,15-TriHETRE";"11,14,15-THETA";"11beta-13,14-dihydro-15-keto PGF2alpha";11-beta-PGE1;11-epi-PGF2alpha;11-epi-Prostaglandin F2alpha;"13,14-dihydro- lipoxin A4";"13,14-dihydro-15-keto PGF2a";"13,14-dihydro-15-keto-PGE1";"13,14-dihydro-15-oxo-PGE1";15-keto-PGF1alpha;15R-PGE1;15R-PGF2alpha;"20-OH-10,11-dihydro-leukotriene B4";6alpha-PGI1;6beta-PGI1;"8-iso-13,14-dihydro-15-keto-PGF2a";8-Isoprostaglandin E1;8-isoprostaglandin PGF2b;"9,11,15-Trihydroxy-prosta-5,13-dien-1-oic acid";PGH1;Prostaglandin D1;Prostaglandin E1;Prostaglandin F2a;Prostaglandin F2alpha;Prostaglandin F2beta;prostaglandin H1 | Oxidised fatty acids |
| 341.2345 | 290 | 0.00162 | 0.46 | methyl 10,13-dihydroxy-9-oxo-11-octadecenoate;"methyl 9,12-dihydroxy-13-oxo-10-octadecenoate";;"(1R,2R)-3-oxo-2-pentyl-cyclopentaneoctanoic acid";"(1S,2S)-3-oxo-2-pentyl-cyclopentaneoctanoic acid";"(9Z)-(12S,13R)-12,13-Epoxyoctadecenoic acid";"(9Z,12Z)-(8S)-Hydroxyoctadeca-9,12-dienoic acid";12(13)-EpOME;"12,13-epoxy-9-octadecenoic acid";12-hydroxy-10-octadecynoic acid;12-hydroxy-9-octadecynoic acid;12-oxo-10E-octadecenoic acid;12-oxo-10Z-octadecenoic acid;12-oxo-9E-octadecenoic acid;12-oxo-9Z-octadecenoic acid;"12R-hydroxy-9Z,15Z-octadecadienoic acid";12S-hydroxy-9-octadecynoic acid;13(R)-HODE;13(S)-HODE;"13-hydroxy-(9Z,11E)-octadecadienoate";"13R-hydroxy-9E,11Z-octadecadienoic acid";"13S-hydroxy-9E,11Z-octadecadienoic acid";18-oxooleate;"6-hydroxy-9Z,12Z-octadecadienoic acid";"8S-hydroxy-9Z,12Z-octadecadienoic acid";9(10)-EpOME;9(R)-HODE;9(S)-HODE;"9,10-epoxy-12-octadecenoic acid";"9,10-epoxyoctadecenoic acid";9-HODE;"9-hydroxy-10E,12Z-octadecadienoic acid";9-hydroxyoctadecadienoate;"9S-hydroxy-10E,12E-octadecadienoic acid | Oxidised fatty acids |
| 383.2159 | 308 | 0.00004 | 0.49 | 10,11-dihydro-12-epi-leukotriene B4;11,12-DHET";11-deoxy-PGE1;11-deoxy-PGF2a;11-deoxy-PGF2beta;12-keto-tetrahydro-Leukotriene B4;"14,15-DHET";"15-hydroperoxyeicosa-8Z,11Z,13E-trienoate";"5,6-DHET";"5,6-dihydroxy-8,11,14-eicosatrienoic acid";"6,7-dihydro-12-epi-leukotriene B4";"8,9-DHET";"8,9-dihydroxy-5,11,14-eicosatrienoic acid | Oxidised fatty acids |
| 189.0765 | 185 | 0.00128 | 0.49 | 3-Hydroxysuberic acid | Oxidised fatty acids |
| 217.1080 | 209 | 0.00034 | 0.51 | Hydroxydecanedioic acid | Oxidised fatty acids |
| 365.1961 | 280 | 0.00071 | 0.52 | 11-dehydro-15-keto-TXB2;11-dehydro-TXB3;"12-oxo-10,11-dihydro-20-COOH-LTB4";12-oxo-20-dihydroxy-leukotriene B4;20-carboxy-leukotriene-B4;20-COOH-Leukotriene B4 | Oxidised fatty acids |
| 383.2427 | 294 | 0.00163 | 0.52 | Dihydroxyoctadecanoic acid | Oxidised fatty acids |
| 278.2317 | 281 | 0.00034 | 0.53 | 3,11-dihydroxy myristoic acid | Oxidised fatty acids |
| 313.1401 | 200 | 0.00295 | 0.54 | Hydroxytetradecanedioic acid | Oxidised fatty acids |
| 228.1599 | 237 | 0.00150 | 0.60 | hydroxy-dodecadienoic acid;oxo-dodecenoic acid | Oxidised fatty acids |
| 299.2577 | 316 | 0.00313 | 0.64 | Hydroxystearate | Oxidised fatty acids |
| 215.0921 | 200 | 0.00122 | 0.65 | 4-Oxosebacic Acid | Oxidised fatty acids |
| 397.1919 | 207 | 0.00313 | 0.66 | 2-HETrE;12R-HETrE;"15-oxo-11Z,13E-eicosadienoic acid";15S-hydroxyeicosatrienoic acid | Oxidised fatty acids |
| 173.1176 | 254 | 0.00182 | 0.69 | hydroxy-nonanoic acid | Oxidised fatty acids |
| 256.1923 | 260 | 0.00389 | 0.73 | hydroxy-tetradecadienoate | Oxidised fatty acids |
| 307.2246 | 309 | 0.00439 | 0.80 | oxo-heptadecanoic acid | Oxidised fatty acids |
| 281.1169 | 190 | 0.00313 | 1.60 | hydroxy-Octadecadienetetraynoic acid | Oxidised fatty acids |
| 234.2058 | 219 | 0.00137 | 1.95 | hydroxy-dodecanoic acid | Oxidised fatty acids |
| 365.2456 | 350 | 0.00122 | 2.02 | hydroxy-eicosenoic acid;oxo-eicosanoic acid | Oxidised fatty acids |
| 339.2288 | 344 | 0.00066 | 2.17 | hydroxy stearic acid | Oxidised fatty acids |
| 313.2755 | 324 | 0.00194 | 3.44 | hydroxy-nonadecanoic acid | Oxidised fatty acids |
| 137.0459 | 64 | 0.00323 | 0.49 | Hypoxanthine | Purine metabolism |
| 375.9961 | 191 | 0.00022 | 28.42 | 5-amino-1-(5-phospho-D-ribosyl)imidazole-4-carboxylate | Purine metabolism |
| 648.3802 | 202 | 0.00361 | 0.01 | N-(tetradecanoyl)-deoxysphing-4-enine-1-sulfonate | Sphingolipids |
| 320.2560 | 299 | 0.00003 | 0.10 | (4E,8Z,d18:2) sphingosine | Sphingolipids |
| 318.2406 | 293 | 0.00003 | 0.25 | (4E,8E,10E-d18:3)sphingosine | Sphingolipids |
| 511.3243 | 309 | 0.00089 | 0.30 | SM(d18:0/0:0) | Sphingolipids |
| 556.3536 | 315 | 0.00005 | 0.32 | N-(2-hydroxyundecanoyl)-4,8-sphingadienine | Sphingolipids |
| 725.5571 | 425 | 0.00028 | 1.53 | SM(d18:1/16:0) | Sphingolipids |
| 402.2591 | 323 | 0.00123 | 1.84 | (4E,8E,9Me-d19:2)sphingosine | Sphingolipids |
| 753.5863 | 449 | 0.00003 | 1.85 | SM(d18:0/18:1) | Sphingolipids |
| 811.6676 | 487 | 0.00150 | 1.86 | SM(d18:0/22:0) | Sphingolipids |
| 831.6337 | 464 | 0.00081 | 1.95 | SM(d18:1/22:0) | Sphingolipids |
| 754.5929 | 449 | 0.00178 | 1.99 | N-(2-hydroxytricosanoyl)-phytosphingosine | Sphingolipids |
| 675.5429 | 406 | 0.00100 | 2.01 | SM(d18:1/14:0) | Sphingolipids |
| 783.6337 | 460 | 0.00221 | 2.11 | SM(d18:0/20:0) | Sphingolipids |
| 849.6097 | 479 | 0.00022 | 2.36 | SM(d18:1/20:0) | Sphingolipids |
| 875.6219 | 483 | 0.00009 | 2.62 | SM(d18:1/22:1) | Sphingolipids |
| 604.3533 | 200 | 0.00021 | 0.01 | all-trans-Hexaprenyl diphosphate | Ubiquinone metabolism and electron transport chain |
| 689.3930 | 337 | 0.00101 | 0.29 | 2-Hexaprenyl-3-methyl-6-methoxy-1,4-benzoquinol | Ubiquinone metabolism and electron transport chain |
| 537.0983 | 273 | 0.00281 | 0.47 | 4-(Cytidine 5'-diphospho)-2-C-methyl-D-erythritol | Ubiquinone metabolism and electron transport chain |
| 284.1136 | 354 | 0.00056 | 0.56 | 5-Hydroxy-2-polyprenylphenol | Ubiquinone metabolism and electron transport chain |
| 771.4842 | 315 | 0.00268 | 0.66 | 2-Octaprenyl-3-methyl-6-methoxy-1,4-benzoquinone | Ubiquinone metabolism and electron transport chain |
| 789.5534 | 435 | 0.00221 | 1.47 | Ubiquinol 8 | Ubiquinone metabolism and electron transport chain |
| 727.5671 | 425 | 0.00066 | 1.50 | Coenzyme Q8 | Ubiquinone metabolism and electron transport chain |
| 838.0496 | 438 | 0.00464 | 1.65 | Coenzyme a Persulfide | Ubiquinone metabolism and electron transport chain |
| 387.0460 | 62 | 0.00439 | 2.10 | 2-Succinyl-5-enolpyruvyl-6-hydroxy-3-cyclohexene-1-carboxylate | Ubiquinone metabolism and electron transport chain |
| 539.2668 | 199 | 0.00003 | 0.02 | 1alpha-hydroxy-22-[3-(1-hydroxy-1-methylethyl)phenyl]-23,24,25,26,27-pentanorvitamin D3 | Vitamin D metabolism |
| 393.2093 | 193 | 0.00003 | 0.07 | (6RS)-22-oxo-23,24,25,26,27-pentanorvitamin D3 6,19-sulfur dioxide adduct | Vitamin D metabolism |
| 577.2666 | 197 | 0.00053 | 0.27 | (17E)-1alpha,25-dihydroxy-26,27-dimethyl-17,20,22,22,23,23-hexadehydro-24a-homovitamin D3;(17Z)-1alpha,25-dihydroxy-26,27-dimethyl-17,20,22,22,23,23-hexadehydro-24a-homovitamin D3;(22E,24E,24bE)-1alpha,25-dihydroxy-22,23,24,24a,24b,24c-hexadehydro-24a,24b,24c-trihomovitamin D3;1alpha,25-dihydroxy-26,27-dimethyl-20,21,22,22,23,23-hexadehydro-24a-homovitamin D3 | Vitamin D metabolism |
| 555.2996 | 203 | 0.00034 | 0.30 | (22E,24E,24bE)-1alpha,25-dihydroxy-26,27-dimethyl-22,23,24,24a,24b,24c-hexadehydro-24a,24b,24c-trihomovitamin D3 | Vitamin D metabolism |
| 637.3479 | 307 | 0.00146 | 0.31 | 1,25-Dihydroxyvitamin D3 3-glycoside | Vitamin D metabolism |
| 599.3097 | 200 | 0.00008 | 0.36 | 26,27-diethyl-1alpha,25-dihydroxy-20,21-didehydro-23-oxavitamin D3 | Vitamin D metabolism |
| 418.3301 | 515 | 0.00268 | 1.74 | 1alpha,25-dihydroxy-23-azavitamin D3;(17E)-1alpha,25-dihydroxy-17,20-didehydro-21-norvitamin D3;(17Z)-1alpha,25-dihydroxy-17,20-didehydro-21-norvitamin D3 | Vitamin D metabolism |
| 367.1138 | 227 | 0.00246 | 4.21 | Alpha-CEHC | Vitamin E metabolism |
| 186.1132 | 203 | 0.00009 | 0.19 | 8-Amino-7-oxononanoate | Vitamin H metabolism |
| 665.3966 | 334 | 0.00257 | 0.35 | Vitamin K2 | Vitamin K metabolism |
