# Supplementary material for: Alterations in bone marrow metabolism are an early and consistent feature during the development of MGUS and multiple myeloma
Source: Blood Cancer J. 2015 Oct 16;5(10):e359–. doi: 10.1038/bcj.2015.85 (PMC4635194; doi:10.1038/bcj.2015.85)
Supplement: Supplementary Table 4 [file bcj201585x5.docx]

**Supplementary Table 4**

Putative metabolites identified in the peripheral plasma that vary significantly (p<0.005) between control and subjects with multiple myeloma

| **m/z** | **Retention time** | **P-value** | **Fold change (control/MM)** | **Metabolite** | **Metabolite class** |
| --- | --- | --- | --- | --- | --- |
| 372.2293 | 193 | 0.00008 | 0.10 | N-Hexadecanoylglycine | Acyl amino acids |
| 447.7623 | 212 | 0.00012 | 0.11 | N-oleoyl taurine | Acyl amino acids |
| 172.0975 | 196 | 0.00100 | 0.43 | Hexanoylglycine;Isovalerylalanine;Isovalerylsarcosine | Acyl amino acids |
| 392.2835 | 454 | 0.00329 | 1.50 | N-stearoyl taurine | Acyl amino acids |
| 240.0993 | 327 | 0.00004 | 1.60 | Capryloylglycine | Acyl amino acids |
| 193.0626 | 199 | 0.00122 | 1.69 | Aminohippuric acid | Acyl amino acids |
| 374.2556 | 252 | 0.00029 | 0.04 | 4,8 dimethylnonanoyl carnitine | Acyl carnitine |
| 340.1883 | 206 | 0.00003 | 0.06 | 2,6 dimethylheptanoyl carnitine | Acyl carnitine |
| 102.1281 | 493 | 0.00389 | 1.46 | Nervonyl carnitine | Acyl carnitine |
| 424.3412 | 293 | 0.00221 | 1.69 | Linoelaidyl carnitine;Linoleyl carnitine | Acyl carnitine |
| 408.3087 | 290 | 0.00043 | 1.80 | pendtadenoyl carnitine | Acyl carnitine |
| 288.2173 | 224 | 0.00122 | 2.33 | Octanoylcarnitine | Acyl carnitine |
| 317.2518 | 247 | 0.00179 | 2.39 | Decanoylcarnitine | Acyl carnitine |
| 481.3487 | 347 | 0.00043 | 0.14 | MG(22:1) | Acyl glycerides |
| 399.2755 | 310 | 0.00049 | 0.27 | MG(18:2) | Acyl glycerides |
| 661.4854 | 344 | 0.00155 | 1.41 | DG(40:10) | Acyl glycerides |
| 465.3917 | 404 | 0.00269 | 1.69 | MG(24:0) | Acyl glycerides |
| 713.5491 | 414 | 0.00313 | 1.81 | DG(40:3) | Acyl glycerides |
| 197.1012 | 40 | 0.00022 | 1.86 | Arginine | Amino acid metabolism |
| 228.0715 | 205 | 0.00323 | 1.94 | Nalpha,Nalpha-Dimethyl-L-histidine | Amino acid metabolism |
| 177.0243 | 39 | 0.00268 | 2.01 | Asparagine | Amino acid metabolism |
| 191.1017 | 221 | 0.00242 | 2.04 | 2,6-Diaminoheptanedioate | Amino acid metabolism |
| 188.0020 | 184 | 0.00310 | 1.58 | Tryptophan | Amino acid metabolism |
| 301.9583 | 44 | 0.00458 | 1.68 | Glutamyl-phosphate | Arginine and proline metabolism |
| 516.3002 | 196 | 0.00005 | 0.04 | Taurocholate;Taurohyocholate;Tauroursocholic acid | Bile acids |
| 575.3170 | 323 | 0.00038 | 0.18 | Lithocholate 3-O-glucuronide | Bile acids |
| 491.2102 | 194 | 0.00003 | 0.03 | Octanoyl-a-D-Glucopyranosyl-B-D-Fructofuranoside | Carbohydrates |
| 271.0400 | 41 | 0.00250 | 1.27 | Hexose sugar | Carbohydrates |
| 306.9749 | 41 | 0.00221 | 1.57 | Ribitol 5-phosphate | Carbohydrates |
| 330.9595 | 184 | 0.00021 | 18.66 | Ribose 1,5-bisphosphate;Ribulose 1,5-bisphosphate | Carbohydrates |
| 832.6639 | 477 | 0.00209 | 1.72 | Galactosylceramide (d18:1/24:1);Glucosylceramide (d18:1/24:1) | Ceramides |
| 806.6373 | 474 | 0.00150 | 1.78 | CerP(d18:1/26:1) | Ceramides |
| 184.0730 | 330 | 0.00389 | 1.45 | Choline phosphate | Choline metabolism |
| 104.1074 | 330 | 0.00028 | 1.55 | Choline | Choline metabolism |
| 435.2234 | 203 | 0.00007 | 0.03 | eicosadienoic acid;Icosatrienoic acid | Fatty acids |
| 371.2199 | 194 | 0.00075 | 0.04 | Eicosapentaenoic acid;Icosapentaenoic acid | Fatty acids |
| 433.2836 | 195 | 0.00072 | 0.06 | Pentacosadiynoic acid | Fatty acids |
| 313.2391 | 279 | 0.00091 | 0.09 | Octadecanedioic acid | Fatty acids |
| 381.1946 | 208 | 0.00003 | 0.32 | Icosatrienoic acid;eicosatrienoic acid | Fatty acids |
| 279.2321 | 301 | 0.00221 | 0.32 | Octadecatrienoic acid | Fatty acids |
| 187.0967 | 220 | 0.00081 | 0.34 | Azelaic acid | Fatty acids |
| 216.1322 | 243 | 0.00123 | 0.53 | Decenoic acid | Fatty acids |
| 159.0655 | 197 | 0.00221 | 0.53 | Dimethylglutaric acid;Methyladipic acid | Fatty acids |
| 247.1191 | 203 | 0.00323 | 0.53 | Sebacic acid | Fatty acids |
| 173.0814 | 209 | 0.00221 | 0.54 | Suberic acid | Fatty acids |
| 315.1547 | 204 | 0.00017 | 0.54 | Tetradecadienoic acid | Fatty acids |
| 199.0973 | 225 | 0.00389 | 0.69 | Decenedioic acid | Fatty acids |
| 316.3211 | 282 | 0.00221 | 1.41 | methyl-octadecanoic acid;Nonadecanoic acid | Fatty acids |
| 253.1410 | 507 | 0.00301 | 1.51 | Dodecanedioic acid | Fatty acids |
| 272.2586 | 316 | 0.00053 | 1.53 | hexadecenoic acid | Fatty acids |
| 274.2739 | 491 | 0.00310 | 1.58 | Hexadecanoate | Fatty acids |
| 477.3918 | 402 | 0.00221 | 1.60 | heptacosenoic acid | Fatty acids |
| 451.3744 | 399 | 0.00467 | 1.61 | Pentacosanoic acid | Fatty acids |
| 172.1334 | 205 | 0.00022 | 1.63 | nonadienoic acid | Fatty acids |
| 415.2723 | 351 | 0.00122 | 1.66 | Docosanoic acid | Fatty acids |
| 247.0239 | 331 | 0.00019 | 1.67 | methyl-hexanoic acid;Heptanoic acid | Fatty acids |
| 455.3842 | 250 | 0.00382 | 1.68 | methyl-hexacosanoic acid;Heptacosanoic acid | Fatty acids |
| 441.1340 | 298 | 0.00022 | 1.74 | Docosatetraynoic acid | Fatty acids |
| 247.0859 | 221 | 0.00323 | 1.74 | methyl nonaoic acid | Fatty acids |
| 260.2588 | 226 | 0.00468 | 1.74 | methyl-tetradecanoic acid;Pentadecanoic acid | Fatty acids |
| 405.2978 | 339 | 0.00043 | 1.77 | docosadienoic acid | Fatty acids |
| 200.1646 | 227 | 0.00034 | 1.78 | Undecanedioic acid | Fatty acids |
| 347.2934 | 354 | 0.00269 | 1.82 | heneicosenoic acid | Fatty acids |
| 269.1272 | 196 | 0.00053 | 1.82 | tridecadienoic acid | Fatty acids |
| 227.1257 | 221 | 0.00066 | 1.87 | Nonanoic acid | Fatty acids |
| 463.3762 | 387 | 0.00044 | 1.98 | hexacosenoic acid | Fatty acids |
| 540.5003 | 339 | 0.00155 | 2.00 | Hexacosanoyl carnitine | Fatty acids |
| 213.1099 | 210 | 0.00043 | 2.04 | Valproic acid | Fatty acids |
| 311.1748 | 323 | 0.00269 | 2.08 | hexadecadienoic acid | Fatty acids |
| 218.2116 | 216 | 0.00028 | 3.06 | Dodecanoate | Fatty acids |
| 259.1996 | 248 | 0.00209 | 1.38 | 1-tetradecanol | Fatty alcohols |
| 355.2818 | 327 | 0.00066 | 1.58 | octadecanediol | Fatty alcohols |
| 373.2629 | 423 | 0.00256 | 1.66 | eicosanol | Fatty alcohols |
| 215.1248 | 199 | 0.00066 | 1.99 | Octanediol | Fatty alcohols |
| 202.2168 | 243 | 0.00281 | 26.98 | dodecenol | Fatty alcohols |
| 304.2610 | 338 | 0.00014 | 0.07 | Elaidoylamide;Oleamide | Fatty amines and amides |
| 256.2634 | 334 | 0.00011 | 0.14 | Palmitic amide | Fatty amines and amides |
| 316.2210 | 287 | 0.00053 | 0.15 | amino-hexadecanoic acid | Fatty amines and amides |
| 348.2876 | 313 | 0.00014 | 0.28 | N-Oleoylethanolamine | Fatty amines and amides |
| 202.1803 | 17 | 0.00458 | 1.50 | amino-undecanoic acid | Fatty amines and amides |
| 376.3187 | 324 | 0.00221 | 1.58 | Anandamide (20:l,n-9) | Fatty amines and amides |
| 374.3031 | 323 | 0.00066 | 1.65 | Anandamide (20:2,n-6) | Fatty amines and amides |
| 222.1827 | 298 | 0.00182 | 1.67 | Dodecanamide | Fatty amines and amides |
| 298.1990 | 228 | 0.00081 | 2.14 | amino-tridecanoic acid | Fatty amines and amides |
| 165.9909 | 530 | 0.00389 | 1.50 | Cysteine | Glutathione metabolism |
| 427.0941 | 51 | 0.00005 | 2.59 | Cysteineglutathione disulfide | Glutathione metabolism |
| 687.3484 | 199 | 0.00017 | 0.38 | Phosphatidate | Glycerophospholipid precursors |
| 216.0628 | 198 | 0.00091 | 3.32 | Glycerylphosphorylethanolamine | Glycerophospholipid precursors |
| 833.4722 | 211 | 0.00005 | 0.04 | PG(38:6) | Glycerophospholipids |
| 855.4819 | 210 | 0.00361 | 0.05 | PG(36:3) | Glycerophospholipids |
| 899.5017 | 211 | 0.00021 | 0.05 | PI(36:2) | Glycerophospholipids |
| 877.4975 | 212 | 0.00008 | 0.08 | PG(34:0) | Glycerophospholipids |
| 943.5329 | 212 | 0.00075 | 0.08 | PIP(36:1) | Glycerophospholipids |
| 863.4423 | 203 | 0.00091 | 0.38 | PE(36:6) | Glycerophospholipids |
| 744.4883 | 415 | 0.00109 | 0.46 | PE(16:1/dm18:1);PE(18:2/dm16:0) | Glycerophospholipids |
| 870.5511 | 459 | 0.00034 | 1.42 | PC(20:2/dm18:1);PC(20:3/dm18:0) | Glycerophospholipids |
| 830.5639 | 430 | 0.00467 | 1.43 | PC(38:5);PC(36:2) | Glycerophospholipids |
| 478.3285 | 432 | 0.00043 | 1.45 | PC(O-16:2/0:0) | Glycerophospholipids |
| 866.5221 | 425 | 0.00122 | 1.49 | PC(20:4/dm18:1);PC(20:5/dm18:0);PC(22:5/dm16:0) | Glycerophospholipids |
| 812.5465 | 430 | 0.00028 | 1.51 | PE(38:4) | Glycerophospholipids |
| 401.2761 | 328 | 0.00022 | 1.54 | PC(O-8:0/O-1:0) | Glycerophospholipids |
| 846.5241 | 419 | 0.00066 | 1.56 | PC(36:6);PC(34:3) | Glycerophospholipids |
| 764.5571 | 445 | 0.00467 | 1.57 | PC(16:1/dm18:1);PC(18:2/dm16:0) | Glycerophospholipids |
| 824.5506 | 457 | 0.00100 | 1.57 | PE(22:4/dm18:0) | Glycerophospholipids |
| 537.1667 | 379 | 0.00150 | 1.64 | PC(12:4) | Glycerophospholipids |
| 825.5604 | 457 | 0.00153 | 1.65 | PG(40:5) | Glycerophospholipids |
| 828.5491 | 422 | 0.00081 | 1.69 | PC(38:6);PC(36:3) | Glycerophospholipids |
| 890.5216 | 422 | 0.00017 | 1.76 | PC(22:6/dm18:1);PE(38:1) | Glycerophospholipids |
| 856.5809 | 445 | 0.00268 | 1.76 | PC(42:9);PC(40:6);PC(38:3) | Glycerophospholipids |
| 814.5555 | 438 | 0.00014 | 1.77 | PE(36:1) | Glycerophospholipids |
| 740.5574 | 462 | 0.00053 | 1.78 | PC(18:3/dm16:0);PC(O-14:0/20:4);PC(14:0/dm18:0);PC(16:0/dm16:0);PC(O-14:0/18:1) | Glycerophospholipids |
| 413.2755 | 459 | 0.00128 | 1.84 | PC(O-10:1/0:0) | Glycerophospholipids |
| 896.5388 | 421 | 0.00323 | 1.94 | PC(38:8);PC(36:5);PC(34:2) | Glycerophospholipids |
| 878.6586 | 267 | 0.00195 | 2.58 | PE(44:2) | Glycerophospholipids |
| 859.4888 | 268 | 0.00451 | 2.64 | PG(40:7) | Glycerophospholipids |
| 555.2680 | 198 | 0.00145 | 0.02 | LysoPG(18:0) | Lysoglycerophospholipids |
| 484.2426 | 199 | 0.00053 | 0.15 | LysoPC(12:0) | Lysoglycerophospholipids |
| 538.2892 | 215 | 0.00008 | 0.26 | LysoPC(18:4);LysoPC(16:1) | Lysoglycerophospholipids |
| 580.2923 | 332 | 0.00091 | 1.51 | LysoPC(dm18:1) | Lysoglycerophospholipids |
| 568.3390 | 327 | 0.00043 | 1.58 | LysoPC(22:6) | Lysoglycerophospholipids |
| 544.3383 | 333 | 0.00389 | 1.63 | LysoPC(20:4) | Lysoglycerophospholipids |
| 610.3097 | 325 | 0.00098 | 1.65 | LysoPC(20:5);LysoPC(18:2) | Lysoglycerophospholipids |
| 542.3223 | 325 | 0.00081 | 1.71 | LysoPC(18:2) | Lysoglycerophospholipids |
| 534.2965 | 332 | 0.00022 | 1.81 | LysoPC(16:0) | Lysoglycerophospholipids |
| 586.3086 | 332 | 0.00011 | 2.15 | LysoPC(18:3) | Lysoglycerophospholipids |
| 227.1035 | 184 | 0.00341 | 0.08 | Prolylhydroxyproline;Methoxytyrosine | Mixed class |
| 360.2766 | 280 | 0.00028 | 0.19 | 12,13-dihydroxy-11-methoxy-9-octadecenoic acid;6-hydroxysphingosine | Mixed class |
| 225.0238 | 574 | 0.00122 | 1.41 | Nicotinurate | NADH metabolism |
| 808.0752 | 448 | 0.00323 | 1.42 | (6S)-6-beta-Hydroxy-1,4,5,6-tetrahydronicotinamide-adenine dinucleotide 2'-phosphate | NADH metabolism |
| 175.0271 | 197 | 0.00071 | 1.86 | 1-Methylnicotinamide | NADH metabolism |
| 369.0067 | 70 | 0.00098 | 0.03 | Pseudouridine;Uridine | Nucleosides and nucleotides |
| 815.0470 | 431 | 0.00081 | 1.49 | Diadenosine triphosphate | Nucleosides and nucleotides |
| 214.9992 | 331 | 0.00014 | 1.61 | Dimethyluracil | Nucleosides and nucleotides |
| 749.5377 | 343 | 0.00057 | 1.39 | CE(20:3) | Other metabolite class |
| 167.0228 | 511 | 0.00122 | 1.42 | Cresol | Other metabolite class |
| 301.0889 | 509 | 0.00389 | 1.50 | Isovalerylglucuronide | Other metabolite class |
| 449.2181 | 209 | 0.00049 | 0.06 | Estradiol-3-glucuronide | Other metabolite class |
| 507.2596 | 211 | 0.00408 | 0.16 | 11-hydroxyprogesterone 11-glucuronide | Other metabolite class |
| 466.3232 | 352 | 0.00014 | 0.29 | 3alpha,6alpha,7alpha,12alpha-Tetrahydroxy-5beta-cholest-24-en-26-oic acid | Other metabolite class |
| 443.3307 | 329 | 0.00034 | 1.65 | (22alpha)-hydroxy-cholestanol;3alpha,7alpha-Dihydroxy-5beta-cholestane;5beta-Cholestane-3alpha,12alpha-diol;5beta-Cholestane-3alpha,26-diol;5-beta-cholestane-3-alpha,7-alpha-diol | Other metabolite class |
| 444.3344 | 329 | 0.00034 | 1.68 | Elocalcitol;;(22alpha)-hydroxy-cholestanol;"3alpha,7alpha-Dihydroxy-5beta-cholestane";"5beta-Cholestane-3alpha,12alpha-diol";"5beta-Cholestane-3alpha,26-diol";"5-beta-cholestane-3-alpha,7-alpha-diol";6alpha-hydroxycholestanol;; | Other metabolite class |
| 225.1083 | 192 | 0.00028 | 2.88 | Dihydrolipoamide | Other metabolite class |
| 298.2472 | 309 | 0.00014 | 0.05 | Epoxyoctadecanoic acid;Hydroxyoctadecenoic acid | Oxidised fatty acids |
| 358.2599 | 272 | 0.00039 | 0.05 | methyl 10,13-dihydroxy-9-oxo-11-octadecenoate;"methyl 9,12-dihydroxy-13-oxo-10-octadecenoate";;"(1R,2R)-3-oxo-2-pentyl-cyclopentaneoctanoic acid";"(1S,2S)-3-oxo-2-pentyl-cyclopentaneoctanoic acid";"(9Z)-(12S,13R)-12,13-Epoxyoctadecenoic acid";"(9Z,12Z)-(8S)-Hydroxyoctadeca-9,12-dienoic acid";12(13)-EpOME;"12,13-epoxy-9-octadecenoic acid";12-hydroxy-10-octadecynoic acid;12-hydroxy-9-octadecynoic acid;12-oxo-10E-octadecenoic acid;12-oxo-10Z-octadecenoic acid;12-oxo-9E-octadecenoic acid;12-oxo-9Z-octadecenoic acid;"12R-hydroxy-9Z,15Z-octadecadienoic acid";12S-hydroxy-9-octadecynoic acid;13(R)-HODE;13(S)-HODE;"13-hydroxy-(9Z,11E)-octadecadienoate";"13R-hydroxy-9E,11Z-octadecadienoic acid";"13S-hydroxy-9E,11Z-octadecadienoic acid";18-oxooleate;"6-hydroxy-9Z,12Z-octadecadienoic acid";"8S-hydroxy-9Z,12Z-octadecadienoic acid";9(10)-EpOME;9(R)-HODE;9(S)-HODE;"9,10-epoxy-12-octadecenoic acid";"9,10-epoxyoctadecenoic acid";9-HODE;"9-hydroxy-10E,12Z-octadecadienoic acid";9-hydroxyoctadecadienoate;"9S-hydroxy-10E,12E-octadecadienoic acid | Oxidised fatty acids |
| 321.2410 | 313 | 0.00007 | 0.08 | (15S)-15-Hydroxy-5,8,11-cis-13-trans-eicosatetraenoate;10-HETE;11(R)-HETE;"11,12:EpETrE";"11,12-EET";"11,12-Epoxyeicosatrienoic acid";"11beta,17beta-Dihydroxy-17-methyl-5alpha-androstan-3-one";11-HETE;"11-hydroxy-5E,8Z,12Z,14Z-eicosatetraenoic acid";11-hydroxyeicosatetraenoate;12 hydroxy arachidonic acid;12-HETE;"12-hydroxy-5Z,8Z,10Z,14Z-Eicosatetraenoic acid";12-hydroxyeicosatetraenoate;"12S-hydroxy-5E,8Z,10Z,14Z-eicosatetraenoic acid";13-HETE;"14,15-EET";"14-hydroxy-5E,8Z,11Z,15Z-eicosatetraenoic acid";"14R,15S-EpETrE";"15(R)-Hydroxy-(5Z,8Z,11Z,13E)-eicosatetraenoate";15-HETE;"15-hydroxy-5E,8Z,11Z,13Z-eicosatetraenoic acid";"15R-hydroxy-5E,8Z,11Z,13Z-eicosatetraenoic acid";16(R)-HETE;17beta-Hydroxy-2alpha-(hydroxymethyl)-5alpha-androstan-3-one;"17beta-Methoxyandrost-5-ene-3beta,16beta-diol";17-HETE;"17-Methylandrost-5-ene-3beta,11beta,17beta-triol";18 hydroxy arachidonic acid;18-HETE;19(S)-HETE;20-HETE;"20-hydroxy-5Z,8Z,11Z,14Z-eicosatetraenoic acid";3beta-Hydroxy-16beta-(hydroxymethyl)-5alpha-androstan-17-one;3R-HETE;"5,6-EET";5-HETE;"6-hydroxy-4E,8Z,11Z,14Z-eicosatetraenoic acid";7-HETE;"8,9-EET";8-HETE;"8-hydroxy-5E,9Z,11Z,14Z-eicosatetraenoic acid";8-hydroxyeicosatetraenoate;"8R-hydroxy-5E,9Z,11Z,14Z-eicosatetraenoic acid";9-HETE;"9R-hydroxy-5E,7Z,11Z,14Z-eicosatetraenoic acid";Hydroxyeicosatetraenoic acid;w-hydroxyl arachidonic acid;;"(6R,7S)-6,7-Epoxyoctadecanoic acid";(9Z)-(12S)-Hydroxyoctadecenoic acid;10-hydroxy-8-octadecenoic acid;10-keto stearic acid;10R-hydroxy-8E-octadecenoic acid;11-hydroxy-9-octadecenoic acid;11-oxo-octadecanoic acid;12-hydroxy-10E-octadecenoic acid;12-oxo-octadecanoic acid;12R-hydroxy-9E-octadecenoic acid;12S-hydroxy-9E-octadecenoic acid;12S-hydroxy-9Z-octadecenoic acid;13-oxo-octadecanoic acid;14-oxo-octadecanoic acid;15-oxo-octadecanoic acid;16-methyl-10-oxo-heptadecanoic acid;16-oxo-octadecanoic acid;17-hydroxy-9Z-octadecenoic acid;17-oxo-octadecanoic acid;18-hydroxy-9Z-octadecenoic acid;18-hydroxyoleate;2-methyl-4-oxo-heptadecanoic acid;2-Oxooctadecanoic acid;3-Oxo-Octadecanoic acid;4-keto stearic acid;5-hydroxy-2-octadecenoic acid;5-keto stearic acid;6-keto stearic acid;7-keto-stearic acid;8-hydroxy-9-octadecenoic acid;8-oxo-octadecanoic acid;8R-hydroxy-9Z-octadecenoic acid;9-hydroxy-10E-octadecenoic acid;9-hydroxy-10Z-octadecenoic acid;9-hydroxy-12-octadecenoic acid;9-hydroxy-12Z-octadecenoic acid;9-keto stearic acid;"9R,10S-epoxy-stearic acid";9R-hydroxy-10E-octadecenoic acid;9R-hydroxy-12E-octadecenoic acid;9R-hydroxy-12Z-octadecenoic acid;"9S,10R-epoxy-stearic acid";Methyl palmoxirate (USAN);Ricinelaidic acid;Ricinoleic acid;Rosaprostol (INN);;14-Methyl-8E-hexadecenal;14R-Methyl-8E-hexadecenal;14R-Methyl-8Z-hexadecenal;14S-Methyl-8E-hexadecenal;14S-Methyl-8Z-hexadecenal;14Z-Methyl-8-hexadecenal;"2,6,8,12-Tetramethyl-2,4-tridecadien-1-ol";9Z-Heptadecenal;; | Oxidised fatty acids |
| 337.2357 | 281 | 0.00316 | 0.10 | (13E)-(15S)-15-Hydroxy-9-oxoprosta-10,13-dienoate;"(13E)-(15S)-15-Hydroxy-9-oxoprosta-11,13-dienoate";"(5Z,8Z,10E,14Z)-(12S)-12-hydroperoxyicosa-5,8,10,14-tetraenoate";"(5Z,8Z,11Z,13E)-(15S)-15-hydroperoxyicosa-5,8,11,13-tetraenoate";"(5Z,9E,11Z,14Z)-(8R)-8-hydroperoxyicosa-5,9,11,14-tetraenoate";"(5Z,9E,14Z)-(8xi,11R,12S)-11,12-Epoxy-8-hydroxyicosa-5,9,14-trienoic acid";"(6E,8Z,11Z,14Z)-(5s)-5-hydroperoxycosa-6,8,11,14-tetraenoate";"(6z,8e,10e,14z)-(5s,12r)-5,12-dihydroxyicosa-6,8,10,14-tetraenoate";"10,11-dihydro-12-oxo-LTB4";11(R)-HPETE;"11,12-DiHETE";"11,12-dihydroxy-5(E),7(E),9(E),14(Z)-eicosatetraenoate";11-deoxy-PGE2;"11H-14,15-EETA";11-HpETE;12(R)-HPETE;12(S)-HPETE;12(S)-Leukotriene B4;"12,20-DiHETE";12R-Hydroperoxyeicosatetraenoate;"14,15-DiHETE";"14,15-dihydroxy-5,8,10,12-eicosatetraenoic acid";"14-hydroperoxy-5Z,8Z,11Z,15E-eicosatetraenoic acid";15(S)-HPETE;15-epi-PGA1;"15H-11,12-EETA";"17,18-DiHETE";20-hydroxy-5S-HETE;"5,12-dihydroxy-6,8,10,14-eicosatetraenoic acid";"5,15-DiHETE";5-HPETE;"5S,15S-DiHETE";"5S,6R-DiHETE";"5S,6S-DiHETE";"6,7-dihydro-5-oxo-12-epi-leukotriene B";6E-12-epi-leukotriene B4;"6-hydroperoxy-4E,8Z,11Z,14Z-eicosatetraenoic acid";6-trans-12-epi-Leukotriene B4;6-trans-Leukotriene B4;8(R)-HPETE;8(S)-HPETE;"8,15-DiHETE";"8,15-dihydroxy-5,9,11,13-eicosatetraenoic acid";"8,9-DiHETE";8-iso-PGA1;"8S,15S-DiHETE";"8S-hydroxy-11R,12S-epoxy-5Z,9E,14Z-eicosatrienoic acid";9(S)-HPETE;9-deoxy-delta12-PGD2;9-HpETE;Diterpenoid SP-II;Hepoxilin A3;Hepoxilin B3;Leukotriene B4;"PGF2alpha-1,11-lactone";"PGF2alpha-1,15-lactone";"PGF2alpha-1,9-lactone";Portulal;Prostaglandin A1;Prostaglandin B1;Prostaglandin C1;;"(9Z)-(7S,8S)-Dihydroxyoctadecenoic acid";"12,13-DHOME";"12,13-dihydroxy-9-octadecenoic acid";"12,13-hydroxyoctadec-9(Z)-enoate";"18-hydroxy-9R,10S-epoxy-stearic acid";"18-hydroxy-9S,10R-epoxy-stearic acid";"7S,8S-dihydroxy-9Z-octadecenoic acid";"9,10-DHOME";"9,10-dihydroxy-12-octadecenoic acid";"9,10-epoxy-18-hydroxystearate";"9,10-hydroxyoctadec-12(Z)-enoate";"9,13-dihydroxy-10-octadecenoic acid";"9,13-dihydroxy-11-octadecenoic acid";Dibutyl sebacate (NF);Octadecanedioic acid;;10E-heptadecenoic acid;10-methyl-9-hexadecenoic acid;10Z-heptadecenoic acid;14-methyl-8E-hexadecenoic acid;14R-methyl-8Z-hexadecenoic acid;14S-methyl-8Z-hexadecenoic acid;16-heptadecenoic acid;2-heptadecylenic acid;2Z-heptadecenoic acid;3-heptadecenoic acid;7-heptadecenoic acid;7-methyl-6E-hexadecenoic acid;7Z-heptadecenoic acid;8E-heptadecenoic acid;8Z-heptadecenoic acid;9E-heptadecenoic acid;9-heptadecylenic acid;omega-Cyclohexylundecanoic acid;; | Oxidised fatty acids |
| 372.2394 | 245 | 0.00232 | 0.12 | methyl 10,12-dihydroperoxy-8E,13E,15Z-octadecatrienoate;"methyl 10,12-epidioxy-13-hydroperoxy-8E,15Z-octadecadienoate";"methyl 10,16-dihydroperoxy-8E,12Z,14E-octadecatrienoate";"methyl 11-(3,5-epidioxy-2-ethyl-cyclopentyl)-9-hydroperoxy-10-undecenoate";"methyl 12,15-epdioxy-16-hydroperoxy-9,13-octadecadienoate";"methyl 13,15-dihydroperoxy-9Z,11E,16E-octadecatrienoate";"methyl 13,15-epidioxy-12-hydroperoxy-9Z,16E-octadecadienoate";"methyl 13,15-epidioxy-16-hydroperoxy-9,11-octadecadienoate";"methyl 13,16-dihydroperoxy-9Z,11E,14E-octadecatrienoate";"methyl 9,12-dihydroperoxy-10E,13E,15Z-octadecatrienoate";"methyl 9,15-dihydroperoxy-10E,12Z,16E-octadecatrienoate";"methyl 9,16-dihydroperoxy-10E,12,14E-octadecatrienoate";"methyl 9-hydroperoxy-10,12-epidioxy-13,15-octadecadienoate";"methyl 9-hydroperoxy-10,13-epdioxy-11,15-octadecadienoate | Oxidised fatty acids |
| 335.2552 | 325 | 0.00017 | 0.12 | oxo-nonadecanoic acid | Oxidised fatty acids |
| 295.2288 | 308 | 0.00122 | 0.20 | hydroxy-tetradecadienoate | Oxidised fatty acids |
| 231.1239 | 220 | 0.00008 | 0.21 | hydroxy-decenoic acid;Oxodecanoate | Oxidised fatty acids |
| 319.2258 | 307 | 0.00034 | 0.23 | (+/-)-11-HEPE;(+/-)-15-HEPE;(+/-)-18-HEPE;(+/-)-8-HEPE;(+/-)-9-HEPE;"(7E,9E,11Z,14Z)-(5S,6S)-5,6-epoxyicosa-7,9,11,14-tetraenoate";11(12)-EpETE;"11-oxo-5E,8Z,12Z,14Z-Eicosatetraenoic acid";11R-HEPE;11S-HEPE;12-HEPE;"12-oxo-5Z,8Z,10E,14Z-eicosatetraenoic acid";12-OxoETE;14(15)-EpETE;15d-PGA1;15-HEPE;"15-oxo-5,8,11-cis-13-trans-icosatetraenoate";15-OxoETE;17(18)-EpETE;;"17R,18S-Epoxy-5Z,8Z,11Z,14Z-icosatetraenoic acid";"4,5-Leukotriene A4";5-HEPE;"5-oxo-(6E,8Z,11Z,14Z)-eicosatetraenoic acid";5-OxoETE;8(9)-EpETE;"8-oxo-5E,9Z,11Z,14Z-eicosatetraenoic acid";8R-HEPE;8S-HEPE;"9-hydroxy-2Z,5E,7Z,11Z,14Z-Eicosapentaenoic acid";"9-oxo-5E,7Z,11Z,14Z-eicosatetraenoic acid";9S-HEPE;"(9Z)-(12S,13R)-12,13-Epoxyoctadecenoic acid";"(9Z,12Z)-(8S)-Hydroxyoctadeca-9,12-dienoic acid";12(13)-EpOME;"12,13-epoxy-9-octadecenoic acid";12-hydroxy-10-octadecynoic acid;12-hydroxy-9-octadecynoic acid;12-oxo-10E-octadecenoic acid;12-oxo-10Z-octadecenoic acid;12-oxo-9E-octadecenoic acid;12-oxo-9Z-octadecenoic acid;"12R-hydroxy-9Z,15Z-octadecadienoic acid";12S-hydroxy-9-octadecynoic acid;13(R)-HODE;13(S)-HODE;"13-hydroxy-(9Z,11E)-octadecadienoate";"13R-hydroxy-9E,11Z-octadecadienoic acid";"13S-hydroxy-9E,11Z-octadecadienoic acid";18-oxooleate;"6-hydroxy-9Z,12Z-octadecadienoic acid";"8S-hydroxy-9Z,12Z-octadecadienoic acid";9(10)-EpOME;9(R)-HODE;9(S)-HODE;"9,10-epoxy-12-octadecenoic acid";"9,10-epoxyoctadecenoic acid";9-HODE;"9-hydroxy-10E,12Z-octadecadienoic acid";9-hydroxyoctadecadienoate;"9S-hydroxy-10E,12E-octadecadienoic acid"; | Oxidised fatty acids |
| 343.1736 | 225 | 0.00049 | 0.28 | 3-Hydroxytetradecanedioic acid | Oxidised fatty acids |
| 411.3084 | 344 | 0.00122 | 0.29 | hydroxy-heneicosanoic acid | Oxidised fatty acids |
| 325.2754 | 322 | 0.00014 | 0.32 | hydroxy-eicosenoic acid;oxo-eicosanoic acid | Oxidised fatty acids |
| 217.1080 | 209 | 0.00034 | 0.39 | Hydroxydecanedioic acid | Oxidised fatty acids |
| 383.2159 | 308 | 0.00081 | 0.42 | 10,11-dihydro-12-epi-leukotriene B4";"11,12-DHET";11-deoxy-PGE1;11-deoxy-PGF2a;11-deoxy-PGF2beta;12-keto-tetrahydro-Leukotriene B4;"14,15-DHET";"15-hydroperoxyeicosa-8Z,11Z,13E-trienoate";"5,6-DHET";"5,6-dihydroxy-8,11,14-eicosatrienoic acid";"6,7-dihydro-12-epi-leukotriene B4";"8,9-DHET";"8,9-dihydroxy-5,11,14-eicosatrienoic acid | Oxidised fatty acids |
| 370.2606 | 291 | 0.00089 | 0.44 | (+/-) 5-iPF2alpha-VI;"(13E)-(15S)-11-alpha,15-dihydroxy-9-oxoprost-13-enoate";"(5Z,13E)-(15S)-9,11,15-trihydroxyprosta-5,13-dienoate";"(5Z,9E,14Z)-(8xi,11xi,12S)-8,11,12-Trihydroxyicosa-5,9,14-trienoate";"11,12,15-THETA";"11,12,15-TriHETRE";"11,14,15-THETA";"11beta-13,14-dihydro-15-keto PGF2alpha";11-beta-PGE1;11-epi-PGF2alpha;11-epi-Prostaglandin F2alpha;"13,14-dihydro- lipoxin A4";"13,14-dihydro-15-keto PGF2a";"13,14-dihydro-15-keto-PGE1";"13,14-dihydro-15-oxo-PGE1";15-keto-PGF1alpha;15R-PGE1;15R-PGF2alpha;"20-OH-10,11-dihydro-leukotriene B4";6alpha-PGI1;6beta-PGI1;"8-iso-13,14-dihydro-15-keto-PGF2a";8-Isoprostaglandin E1;8-isoprostaglandin PGF2b;"9,11,15-Trihydroxy-prosta-5,13-dien-1-oic acid";PGH1;Prostaglandin D1;Prostaglandin E1;Prostaglandin F2a;Prostaglandin F2alpha;Prostaglandin F2beta;prostaglandin H1 | Oxidised fatty acids |
| 330.2378 | 258 | 0.00043 | 0.44 | trihydroxy-octadecenoic acid | Oxidised fatty acids |
| 278.2317 | 281 | 0.00221 | 0.45 | 3,11-dihydroxy myristoic acid | Oxidised fatty acids |
| 315.2550 | 294 | 0.00182 | 0.51 | Dihydroxyoctadecanoic acid | Oxidised fatty acids |
| 299.2577 | 316 | 0.00182 | 0.57 | Hydroxystearate | Oxidised fatty acids |
| 379.2452 | 304 | 0.00028 | 0.59 | 13,14-dihydro PGE1;"13,14-dihydro PGF2a";"13,14-dihydro-15-keto-PGF1alpha";"13,14-dihydro-PGE1";"13,14-dihydro-PGF2alpha";PGF1beta;Prostaglandin F1alpha | Oxidised fatty acids |
| 302.2691 | 286 | 0.00182 | 1.45 | oxo-heptadecanoic acid | Oxidised fatty acids |
| 249.0732 | 510 | 0.00467 | 1.49 | hydroxy-Decadienediynoic acid | Oxidised fatty acids |
| 299.0735 | 510 | 0.00467 | 1.50 | 4,7-Dioxosebacic Acid | Oxidised fatty acids |
| 404.2772 | 290 | 0.00182 | 1.62 | 11,12-DiHETrE-EA;"14,15-DiHETrE-EA";"5,6-DiHETrE-EA";"8,9-DiHETrE-EA | Oxidised fatty acids |
| 331.2231 | 319 | 0.00014 | 1.63 | methyl 12,13-epoxy-9,15-octadecadienoate;"methyl 15,16-epoxy-9,12-octadecadienoate";"methyl 9,10-epoxy-12,15-octadecadienoate | Oxidised fatty acids |
| 339.2288 | 344 | 0.00382 | 1.82 | hydroxy stearic acid | Oxidised fatty acids |
| 232.1905 | 242 | 0.00014 | 1.85 | hydroxy-dodecenoic acid;oxo-dodecanoic acid | Oxidised fatty acids |
| 290.2689 | 491 | 0.00038 | 1.86 | hydroxy-hexadecanoic acid | Oxidised fatty acids |
| 287.2211 | 311 | 0.00182 | 1.94 | hydroxy-oxo-hexadecanoic acid;Hexadecanedioic acid | Oxidised fatty acids |
| 386.2668 | 428 | 0.00004 | 1.95 | 11(12)-EpETrE-EA;14(15)-EpETrE-EA;5(6)-EpETrE-EA;8(9)-EpETrE-EA;Leukotriene B4 dimethylamide | Oxidised fatty acids |
| 262.2379 | 217 | 0.00022 | 2.18 | hydroxy-tetradecanoic acid | Oxidised fatty acids |
| 234.2058 | 219 | 0.00028 | 2.60 | hydroxy-dodecanoic acid | Oxidised fatty acids |
| 313.2755 | 324 | 0.00047 | 7.05 | hydroxy-nonadecanoic acid | Oxidised fatty acids |
| 290.1065 | 201 | 0.00028 | 9.65 | Hydroxydodecanedioic acid | Oxidised fatty acids |
| 343.0638 | 513 | 0.00382 | 1.42 | 7-Methylxanthosine;Deoxyinosine | Purine metabolism |
| 271.0422 | 511 | 0.00464 | 1.50 | 1,7-Dimethylxanthine;Theobromine;Theophylline | Purine metabolism |
| 133.0347 | 194 | 0.00433 | 1.73 | N,N'-Dimethylurea | Purine metabolism |
| 375.9961 | 191 | 0.00012 | 46.02 | 5-amino-1-(5-phospho-D-ribosyl)imidazole-4-carboxylate | Purine metabolism |
| 648.3802 | 202 | 0.00408 | 0.01 | N-(tetradecanoyl)-deoxysphing-4-enine-1-sulfonate | Sphingolipids |
| 320.2560 | 299 | 0.00007 | 0.05 | (4E,8Z,d18:2) sphingosine | Sphingolipids |
| 556.3536 | 315 | 0.00022 | 0.19 | N-(2-hydroxyundecanoyl)-4,8-sphingadienine | Sphingolipids |
| 318.2406 | 293 | 0.00007 | 0.21 | (4E,8E,10E-d18:3)sphingosine | Sphingolipids |
| 511.3243 | 309 | 0.00111 | 0.24 | SM(d18:0/0:0) | Sphingolipids |
| 384.2716 | 428 | 0.00122 | 1.49 | 6-hydroxysphingosine | Sphingolipids |
| 410.3244 | 287 | 0.00081 | 1.53 | N,N,N-trimethyl-sphingosine | Sphingolipids |
| 318.3001 | 490 | 0.00208 | 1.58 | 4-hydroxysphinganine | Sphingolipids |
| 725.5571 | 425 | 0.00034 | 1.58 | SM(d18:1/16:0) | Sphingolipids |
| 753.5863 | 449 | 0.00017 | 1.65 | SM(d18:0/18:1) | Sphingolipids |
| 402.2591 | 323 | 0.00372 | 1.67 | (4E,8E,9Me-d19:2)sphingosine | Sphingolipids |
| 622.5410 | 405 | 0.00389 | 1.67 | N-(2-hydroxyoctadecanoyl)-phytosphingosine | Sphingolipids |
| 754.5929 | 449 | 0.00086 | 2.19 | N-(2-hydroxytricosanoyl)-phytosphingosine | Sphingolipids |
| 604.3533 | 200 | 0.00028 | 0.02 | all-trans-Hexaprenyl diphosphate | Ubiquinone metabolism and electron transport chain |
| 771.4842 | 315 | 0.00389 | 0.65 | 2-Octaprenyl-3-methyl-6-methoxy-1,4-benzoquinone | Ubiquinone metabolism and electron transport chain |
| 789.5534 | 435 | 0.00268 | 1.39 | Ubiquinol 8 | Ubiquinone metabolism and electron transport chain |
| 727.5671 | 425 | 0.00043 | 1.51 | Coenzyme Q8 | Ubiquinone metabolism and electron transport chain |
| 345.0794 | 508 | 0.00221 | 1.60 | Thiamin Phosphate | Vitamin B ,etabolism |
| 539.2668 | 199 | 0.00009 | 0.01 | 1alpha-hydroxy-22-[3-(1-hydroxy-1-methylethyl)phenyl]-23,24,25,26,27-pentanorvitamin D3 | Vitamin D metabolism |
| 531.2809 | 209 | 0.00075 | 0.04 | 1,25-dihydroxy-2-nor-1,2-secovitamin D3;1alpha,25-dihydroxy-19-norvitamin D3;2-Nor-1,3-seco-1alpha,25-dihydroxyvitamin D3 | Vitamin D metabolism |
| 394.2129 | 193 | 0.00003 | 0.06 | (6RS)-22-oxo-23,24,25,26,27-pentanorvitamin D3 6,19-sulfur dioxide adduct | Vitamin D metabolism |
| 393.2093 | 193 | 0.00003 | 0.06 | (6RS)-22-oxo-23,24,25,26,27-pentanorvitamin D3 6,19-sulfur dioxide adduct | Vitamin D metabolism |
| 555.2996 | 203 | 0.00005 | 0.18 | (22E,24E,24bE)-1alpha,25-dihydroxy-26,27-dimethyl-22,23,24,24a,24b,24c-hexadehydro-24a,24b,24c-trihomovitamin D4 | Vitamin D metabolism |
| 599.3097 | 200 | 0.00007 | 0.27 | 26,27-diethyl-1alpha,25-dihydroxy-20,21-didehydro-23-oxavitamin D3 | Vitamin D metabolism |
| 637.3479 | 307 | 0.00024 | 0.31 | 1,25-Dihydroxyvitamin D3 3-glycoside | Vitamin D metabolism |
| 375.2535 | 319 | 0.00268 | 1.39 | 1alpha-hydroxy-24,25,26,27-tetranorvitamin D3 23-carboxylic acid | Vitamin D metabolism |
| 529.3862 | 383 | 0.00120 | 1.54 | 24-Hydroxy-19-norgeminivitamin D3 | Vitamin D metabolism |
| 417.2981 | 335 | 0.00208 | 2.52 | (24R,25S)-25,26-epoxy-1alpha,24-dihydroxy-27-norvitamin D3;(24S,25R)-25,26-epoxy-1alpha,24-dihydroxy-27-norvitamin D3;"1alpha,25-Dihydroxy-16-ene-19-nor-24-oxovitamin D3 | Vitamin D metabolism |
| 186.1132 | 203 | 0.00005 | 0.10 | 8-Amino-7-oxononanoate | Vitamin H metabolism |
| 665.3966 | 334 | 0.00489 | 0.28 | Vitamin K2 | Vitamin K metabolism |
