# Supplementary material for: Alterations in bone marrow metabolism are an early and consistent feature during the development of MGUS and multiple myeloma
Source: Blood Cancer J. 2015 Oct 16;5(10):e359–. doi: 10.1038/bcj.2015.85 (PMC4635194; doi:10.1038/bcj.2015.85)
Supplement: Supplementary Table 5 [file bcj201585x6.docx]

**Supplementary Table 5**

Putative metabolites identified in the peripheral plasma that vary significantly (p<0.005) between subjects with MGUS and those with multiple myeloma

| **m/z** | **Retention time** | **P-value** | **Fold change (MGUS/MM)** | **Metabolite** | **Metabolite class** |
| --- | --- | --- | --- | --- | --- |
| 229.1449 | 254 | 0.001451 | 1.51 | Dodecanedioic acid | Fatty acids |
| 347.2194 | 302 | 0.000451 | 1.39 | octadecatrienoic acid | Fatty acids |
| 259.1996 | 248 | 0.002089 | 1.49 | 1-tetradecanol | Fatty alcohols |
| 753.5215 | 413 | 0.003892 | 0.55 | PC(32:2) | Glycerophospholipids |
| 856.6097 | 464 | 0.00268 | 0.55 | PC(38:3) | Glycerophospholipids |
| 898.5701 | 417 | 0.004669 | 1.65 | PC(42:7) | Glycerophospholipids |
| 862.4362 | 203 | 0.003477 | 0.63 | PE(36:6) | Glycerophospholipids |
| 538.3172 | 320 | 0.003892 | 0.41 | LysoPC(16:1) | Lysoglycerophospholipids |
| 706.3459 | 348 | 0.000889 | 0.58 | LysoPC(18:0) | Lysoglycerophospholipids |
| 590.3463 | 330 | 0.003235 | 0.49 | LysoPC(20:3) | Lysoglycerophospholipids |
| 588.3333 | 323 | 0.003892 | 0.48 | LysoPC(20:4) | Lysoglycerophospholipids |
| 526.3124 | 323 | 0.004669 | 0.46 | LysoPE(18:0) | Lysoglycerophospholipids |
| 562.0256 | 418 | 0.00281 | 1.91 | CDP-glycerol | Nucleosides and nucleotides |
| 716.2674 | 317 | 0.002953 | 0.30 | 5-Methyltetrahydropteroyltri-L-glutamate | Other metabolite class |
| 628.2256 | 354 | 0.002249 | 2.05 | Dehydroisocoproporphyrinogen | Other metabolite class |
| 228.1599 | 237 | 0.003235 | 1.88 | hydroxy-dodecadienoic acid;oxo-dodecenoic acid | Oxidised fatty acids |
| 286.2138 | 295 | 0.003235 | 1.41 | hydroxy-oxo-hexadecanoic acid;Hexadecanedioic acid | Oxidised fatty acids |
| 201.1493 | 273 | 0.000429 | 1.47 | hydroxy-undecanoic acid | Oxidised fatty acids |
| 239.0167 | 47 | 0.004284 | 2.49 | Dimethyluric acid | Purine metabolism |
| 283.1037 | 220 | 0.00128 | 4.82 | Methylinosine | Purine metabolism |
| 290.9866 | 268 | 0.003445 | 2.03 | Methylxanthine | Purine metabolism |
| 875.6219 | 483 | 0.003235 | 0.56 | SM(d18:1/22:1) | Sphingolipids |
| 191.0191 | 176 | 0.001372 | 1.81 | Citrate;Isocitrate | TCA cycle |
| 537.0983 | 273 | 0.00382 | 2.15 | 4-(Cytidine 5'-diphospho)-2-C-methyl-D-erythritol | Ubiquinone metabolism and electron transport chain |
| 284.1136 | 354 | 0.003716 | 1.48 | 5-Hydroxy-2-polyprenylphenol | Ubiquinone metabolism and electron transport chain |
| 994.5934 | 467 | 0.00281 | 0.51 | All trans decaprenyl diphosphate | Ubiquinone metabolism and electron transport chain |
