# Supplementary material for: Alterations in bone marrow metabolism are an early and consistent feature during the development of MGUS and multiple myeloma
Source: Blood Cancer J. 2015 Oct 16;5(10):e359–. doi: 10.1038/bcj.2015.85 (PMC4635194; doi:10.1038/bcj.2015.85)
Supplement: Supplementary Methods [file bcj201585x7.docx]

**Methods**

**Sample collection, preparation and patient characteristics**

Patients with MM and MGUS were recruited from specialist clinics at The Royal Wolverhampton Hospitals NHS Trust and Heart of England NHS Trust, UK. The study received approval from the local ethics committees at South Birmingham, Birmingham East, North, and Solihull (West Midlands, UK), and informed written consent was obtained in accordance with the Declaration of Helsinki in all cases. Two groups of ‘control’ subjects were used in this study. The first, used for the investigation of the bone marrow metabolome consisted of 10 patients undergoing thoracotomies: around 80% of the patients recruited to this study as a control group had a primary lung tumor. As it was possible that the peripheral blood of these patients may show metabolic changes, a second group of 11 healthy controls were used for the investigation into the peripheral plasma metabolome, consisting of clinically healthy age-matched individuals with no long term conditions and not on any medication that could modify immunity.

For peripheral blood analysis, samples were obtained from patients at diagnosis into lithium/heparin tubes, placed on ice for transport to the laboratory, layered over Lymphoprep (5 mL/15mL diluted blood) after 1:1 dilution in RPMI (Life Technologies, UK) and centrifuged to remove cellular components. In total, samples from 12 MGUS and 11 MM patients were collected and analysed. Resulting diluted plasma was snap frozen at -80°C. Collection and analysis of bone marrow aspirates was similar, except that the initial dilution of sample in Lymphoprep was 1:2. For the bone marrow study, samples from 10 patients with MGUS and MM were collected and analysed. For all samples, time from collection to freezing was kept to less than 6 hours and all processes and storage were performed on ice or cooled to 4^o^C to minimise metabolic changes in the sample. Patient characteristics are shown in Supplementary Tables 1 and 2. For both analytical methods below, samples collected were first filtered by loading into Nanosep tubes (VWR, UK) before centrifuging at 14000 x g for 20 minutes at 4 ^o^C.

**NMR spectroscopy**

The filtered plasma was then mixed with phosphate buffer (pH 7.0), TMSP [(3-trimethylsilyl)-propionic-(2,2,3,3-d_4_) acid, sodium salt] and D_2_O to a final concentration of 100 mM, 500 μM and 10% respectively. ^1^H-NMR spectra were acquired using a Bruker AVII 500 MHz spectrometer equipped with a 5 mm inverse Cryoprobe with a sample temperature of 300 K using NOESY-presat to suppress the water resonance ([1](#_ENREF_1)). 1D-NMR spectra were acquired using a 7194 Hz spectral width (12 ppm), 16384 complex data points, 4 second relaxation delay, 16 steady state scans and 256 transients. The probe was manually tuned and matched prior to acquisition of first sample and each sample was automatically shimmed using one-dimensional gradient shimming to a TMSP line width of less than 1 Hz prior to acquisition of each sample. All data sets were processed using the MATLAB based MetaboLab software ([2](#_ENREF_2)). Spectra were manually phase corrected, followed by baseline correction using a spline function to model the baseline. The water region, low field area of the spectrum (>9.5ppm) and region between 0.05 ppm and the right hand end of the spectrum were excluded. In order to account for small chemical shift variations between the different samples segmental alignment of all resonances was performed using Icoshift ([3](#_ENREF_3)) and then scaled with respect to each other using the probabilistic quotient normalization ([4](#_ENREF_4)). For variance stabilization NMR spectra were then transformed using the extended glog transform with λ=1e-11 and y0=5e^-5^ ([5](#_ENREF_5)). Spectra were then exported into PLS-toolbox as a data set object for statistical analysis. Principal component analysis (PCA), an unbiased multivariate analysis method in which samples are clustered based on the similarity of their spectra or separated based on the dissimilarity of their spectra was used to analyse the resulting spectra. In order to quantify those metabolites that contributed to the separation of the patient groups, the metabolite peaks that contributed to the loadings of PC1 (i.e. those metabolite peaks that allowed differentiation of disease from non-disease-associated bone marrow) were identified. As the peaks themselves shown in the loading plots are not dependent on the metabolite concentrations *per se* but the degree of change between the different groups measured, we then used the origina­l spectra to calculate the absolute concentrations of the metabolites identified in each group. Kruskal-Wallis tests were used to test for significant changes in metabolite concentrations between groups, and where a significant change was observed, a Dunn’s multiple comparisons post-hoc test was performed where shown.

**Ultra High Performance Liquid Chromatography-Mass Spectrometry**

All solvents and chemicals applied were of HPLC analytical grade (J.T. Baker, U.K.).

Plasma samples were prepared as previously described ([6](#_ENREF_6)). In summary, 600 µL of methanol was added to 200 µL of plasma, vortex mixed for 15 seconds followed by centrifugation (13,000 *g*, 15 minutes). Two 370 µL aliquots were transferred to separate 1.5mL tubes, dried by speed vacuum concentration at room temperature for 5 hours (Thermo Savant SPD111V Modular Concentrator and RVT4 104 cooled solvent trap) and stored at -80^o^C. A single pooled QC sample was prepared by mixing 100 µL of plasma from each subject and preparing separate QC samples as described above. Samples were reconstituted in 100 µL of 50:50 methanol:water, vortex mixed and centrifuged for 15 min at 10 000g and transferred to vials with 200 mL fixed inserts (Thermo-Fisher Ltd, U.K.). All samples were stored in the autosampler at 5°C and analysed separately in negative and positive electrospray ionisation (ESI) modes within 72 h of reconstitution. UHPLC separations were performed applying a Hypersil Gold C18 reversed phase column (100 x 2.1 mm, 1.9 mm) at a flow rate of 400 µL min^-1^, temperature of 40°C and with two solvents: solvent A (HPLC grade water +0.1% formic acid) and solvent B (HPLC grade methanol +0.1% formic acid). A gradient elution was performed as follows: hold 100% A 0–1.5 min, 100% A–100% B 1.5–6 min curve 3, hold 100% B 6–12 min, 100% B–100% A 12–13 min curve 3, hold 100% A 13–15 min. Injection volume was 5 µL. UHPLC eluent was introduced directly in to the electrospray LTQ-FT Ultra mass spectrometer with source conditions as follows: spray voltage -4.5 kV (ESI-) and +5 kV (ESI+), sheath gas 30 arbitrary units, aux gas 15 arbitrary units, capillary voltage 35 V, tube lens voltage -100 V (ESI-) and +90 V (ESI+), capillary temperature 280°C, ESI heater temperature 300°C. Data were acquired in the FT mass spectrometer in the m/z range 100–1000 at a mass resolution of 50 000 (FWHM defined at *m/z* 400), with a scan speed of 0.4 s and an AGC setting of 1x10^6^. Analysis order was composed of 10 QC sample injections for system conditioning followed by a QC sample injection every 6th injection with two QC sample injections at the end of the analytical run. Plasma extracts were analysed in a random order. UHPLC-MS raw data profiles were first converted into a NetCDF format within the Xcalibur software’s File Converter program. Each NetCDF based three-dimensional data matrix (intensity x m/z x time – one per sample) was converted (or deconvolved) into a vector of peak responses, where a peak response is defined as the sum of intensities over a window of specified mass and time range (e.g. m/z = 102.1 +/- 0.01 and time = 130 +/- 10 s). In this experiment the deconvolution was performed using the freely available XCMS software as described previously [2]. Data were exported from XCMS as a .csv file for further data analysis. The quality of data was assessed applying QC data as previously described [3] with all metabolite features with a RSD > 20% for QC samples being removed from the dataset prior to data analysis. The data for each sample was normalised (as a percentage) to the total peak area for all metabolites in the sample and glog scaling was applied. Metabolite annotation was performed applying the PUTMEDID_LCMS workflow as previously described.([7](#_ENREF_7)) All metabolite annotations are reported at level 2 (putatively annotated compounds) according to MSI reporting standards.([8](#_ENREF_8)) In cases where a single metabolite is detected as multiple metabolite features (as described previously([9](#_ENREF_9))), only a single feature is reported chosen as having the smallest critical p-value. The processed data were analysed in R applying the unsupervised multivariate principal components analysis (PCA) and the univariate non-parametric Wilcoxon–Mann–Whitney test. No correction for false discovery rate was applied. The fold change (median class 1/median class 2) was calculated. Metabolites were manually clustered in to classes defining similar chemical structure or metabolic pathway to identify biologically relevant and robust metabolic changes.

**SUPPLEMENTARY REFERENCES**

1. Gueron M, Plateau P, Decorps M. Solvent Signal Suppression in Nmr. Prog Nucl Mag Res Sp. 1991;23:135-209.

2. Ludwig C, Gunther UL. MetaboLab - advanced NMR data processing and analysis for metabolomics. Bmc Bioinformatics. 2011;12.

3. Savorani F, Tomasi G, Engelsen SB. icoshift: A versatile tool for the rapid alignment of 1D NMR spectra. J Magn Reson. 2010;202(2):190-202.

4. Dieterle F, Ross A, Schlotterbeck G, Senn H. Probabilistic quotient normalization as robust method to account for dilution of complex biological mixtures. Application in H-1 NMR metabonomics. Anal Chem. 2006;78(13):4281-90.

5. Parsons HM, Ludwig C, Gunther UL, Viant MR. Improved classification accuracy in 1- and 2-dimensional NMR metabolomics data using the variance stabilising generalised logarithm transformation. Bmc Bioinformatics. 2007;8:234.

6. Dunn WB, Broadhurst D, Begley P, Zelena E, Francis-McIntyre S, Anderson N, et al. Procedures for large-scale metabolic profiling of serum and plasma using gas chromatography and liquid chromatography coupled to mass spectrometry. Nature protocols. 2011;6(7):1060-83.

7. Brown M, Wedge DC, Goodacre R, Kell DB, Baker PN, Kenny LC, et al. Automated workflows for accurate mass-based putative metabolite identification in LC/MS-derived metabolomic datasets. Bioinformatics. 2011;27(8):1108-12.

8. Sumner LW, Amberg A, Barrett D, Beale MH, Beger R, Daykin CA, et al. Proposed minimum reporting standards for chemical analysis Chemical Analysis Working Group (CAWG) Metabolomics Standards Initiative (MSI). Metabolomics. 2007;3(3):211-21.

9. Brown M, Dunn WB, Dobson P, Patel Y, Winder CL, Francis-McIntyre S, et al. Mass spectrometry tools and metabolite-specific databases for molecular identification in metabolomics. The Analyst. 2009;134(7):1322-32.
